# Exploring the Therapeutic Potential of Lycopene: Mechanisms, Biological Activities, and Health Benefits

**DOI:** 10.3390/ijms27125386

**Published:** 2026-06-15

**Authors:** Saleh A. Almatroodi, Mohammad Alshebremi, Arshad Husain Rahmani

**Affiliations:** Department of Medical Laboratories, College of Applied Medical Sciences, Qassim University, Buraydah 51452, Saudi Arabia; smtrody@qu.edu.sa (S.A.A.); m.alshebremi@qu.edu.sa (M.A.)

**Keywords:** lycopene, anti-inflammatory activity, antioxidant activity, chronic diseases, cancer, nanoformulation

## Abstract

Lycopene, a carotenoid commonly found in tomatoes, is an emerging compound with a noteworthy role in the prevention and treatment of various pathologies. Evidence from in vitro and in vivo models indicates that lycopene plays an imperative role in disease management through the regulation of oxidative stress, inflammation, and the modulation of various biological processes. Its role containing nephroprotective, hepatoprotective, cardioprotective, and neuroprotective properties, anti-arthritis, anti-obesity, and wound healing has been documented. Its significant role in cancer management through modulation of inflammation, angiogenesis, apoptosis, cell cycle, and PI3/Akt pathways has been properly described. This review aims to provide a comprehensive overview of the role of lycopene in the management of pathogenesis. The review further evaluates the current evidence regarding the synergistic interactions of lycopene with other bioactive compounds and drugs and highlights the potential of emerging nanoformulation strategies to improve its bioavailability and therapeutic efficacy in disease management. However, this compound shows some limitations, particularly its low bioavailability and poor water solubility, which hinder its therapeutic benefit in disease management. For a better understanding of its mechanisms of action, safety considerations, potential drug interactions, and optimal dosages, further detailed studies based on both preclinical and clinical data are needed. This further investigation is essential to reveal the maximum potential benefit of lycopene in disease management.

## 1. Introduction

Medicinal herbs have served as the basis of alternative medicine and have provided the primary pathway for developing novel drugs [1]. Moreover, flavonoids are natural compounds in various plants, leaves, fruits, and flowers. Its role in disease prevention and treatment has been reported mainly through the modulation of antioxidant, anti-inflammatory, and cellular and molecular pathways [2,3,4].

Lycopene is a carotenoid with molecular formula C40H56 and molecular weight 536.85 g/mol. Its relative molecular mass is 536.85 g/mol. It is primarily found in its trans-isomer form in different amounts in tomatoes and is also present in other red fruits and vegetables like red carrots, grapefruits, watermelons, and papayas. Its significant role in the management of various pathogeneses has been proven through different mechanisms. Its capability to prevent disease mainly arises from its antioxidant and anti-inflammatory properties [5]. A growing body of evidence suggests that lycopene may play a protective role in the prevention and management of various diseases by modulating oxidative stress, inflammation, apoptosis, and related molecular pathways. A study was performed to confirm the supplementation effect of tomato juice on indices linked with metabolic health as well as adipokine profiles in generally healthy people. The finding reported that supplementation of tomato juice reduced the inflammatory adipokine MCP-1 whereas anti-inflammatory adiponectin levels increasing in healthy Taiwanese females aged 20–30 years [6]. The role of lycopene supplementation on endothelial function is measured through reactive hyperemia peripheral arterial tonometry and oxidative stress. Study based on findings concluded that increase in serum lycopene after supplementation decreases oxidative stress which might show a role in endothelial function [7]. Lycopene has demonstrated the ability to reverse reactive oxygen species (ROS)-mediated alterations in the motility, viability, and antioxidant profile of bovine spermatozoa exposed to ferrous ascorbate (FeAA). Treatment with FeAA meaningfully reduced sperm motility, viability, as well as antioxidant capacity, while increasing superoxide production, ROS generation, and lipid peroxidation. In contrast, lycopene administration preserved sperm motility parameters, preserved mitochondrial activity, and enhanced the antioxidant status of spermatozoa [8]. Protective activity of topical lycopene in acute ultraviolet B (UVB)-induced photodamage was investigated. Lycopene applications dependently inhibited UVB-induced myeloperoxidase as well as ornithine decarboxylase and bifold skin thickness reduced. The topical lycopene application inhabited the cleavage of caspase-3 and reversed UVB-induced PCNA inhibition. These outcomes recommend that lycopene topical is capable to exert its effects in acute UVB-induced photodamage [9]. Tarfa Albrahim, 2022, performed a study to investigate the role of lycopene in an animal model of hypercholesterolemia [10]. The study reported that rats showed elevated serum lipid levels, marked oxidative stress, renal and cardiac dysfunction, and increased apoptotic and pro-inflammatory markers by the end of the experiment. Treatment by lycopene significantly ameliorated and restored these alterations [10]. The study explored the neuroprotective and antioxidant effects of lycopene and insulin on passive avoidance memory, malondialdehyde (MDA) levels, total antioxidant capacity (TAC), and apoptosis inhibition in the hippocampus of streptozotocin-induced diabetic rats. The results exhibited that lycopene and insulin, either alone or combined, reduced hippocampal neuronal cell death and enhanced learning and cognitive function by decreasing MDA levels and increasing TAC levels [11].

Although earlier reviews have described the health benefits of lycopene, there is also dearth in critical evaluation of its mechanisms, synergistic interactions, and formulation-related challenges. Therefore, this review provides a focused overview of lycopene by summarizing its dietary sources, mechanisms in disease modulation, synergistic effects with other compounds and drugs, and the role of nanoformulations in improving bioavailability and therapeutic efficacy. In addition, tabulated summaries of doses, experimental models, and therapeutic outcomes across different pathological conditions are included, offering a more comprehensive perspective on lycopene in disease management.

## 2. Methodology

The wide-ranging literature was searched to identify appropriate studies exploring lycopene and its potential role in different pathogenesis. The electronic databases, such as Scopus, PubMed, Google, and Google Scholar, were searched to confirm comprehensive coverage of the available literature to compile the data. The included studies in this review were selected based on the references published between January 2000 and March 2026. The search approach employed the following: “lycopene and antioxidant”, “lycopene” and anti-inflammatory”, “lycopene and cardioprotective”, “lycopene and neuroprotective”, “lycopene and anti-diabetic”, “lycopene and anti-obesity”, “lycopene and hepatoprotective”, “lycopene and respiratory diseases”, “lycopene and digestive system”, “lycopene and cancer”, “lycopene and antimicrobial”, “lycopene and nanoformulation”, “lycopene synergistic effects”, “lycopene and antifungal”, and “lycopene and clinical trials”. Additional searches included terms associated with dietary sources, daily intake, bioavailability, and the mechanisms of action of lycopene. At first, 245 publications were checked and after removing duplicate studies, 219 publications were included in the review. Research articles, review articles, as well as human clinical studies focusing on biological activities, mechanisms of action, and lycopene disease-management applications were included. Studies evaluating lycopene in preclinical models, human clinical trials, and nanoformulation-based approaches were also considered. Case reports, editorials, conference abstracts and non-English publications were excluded. Duplicates or substantially similar publications were also excluded.

## 3. Lycopene Structure, Sources and Daily Intake

**Figure 1:**
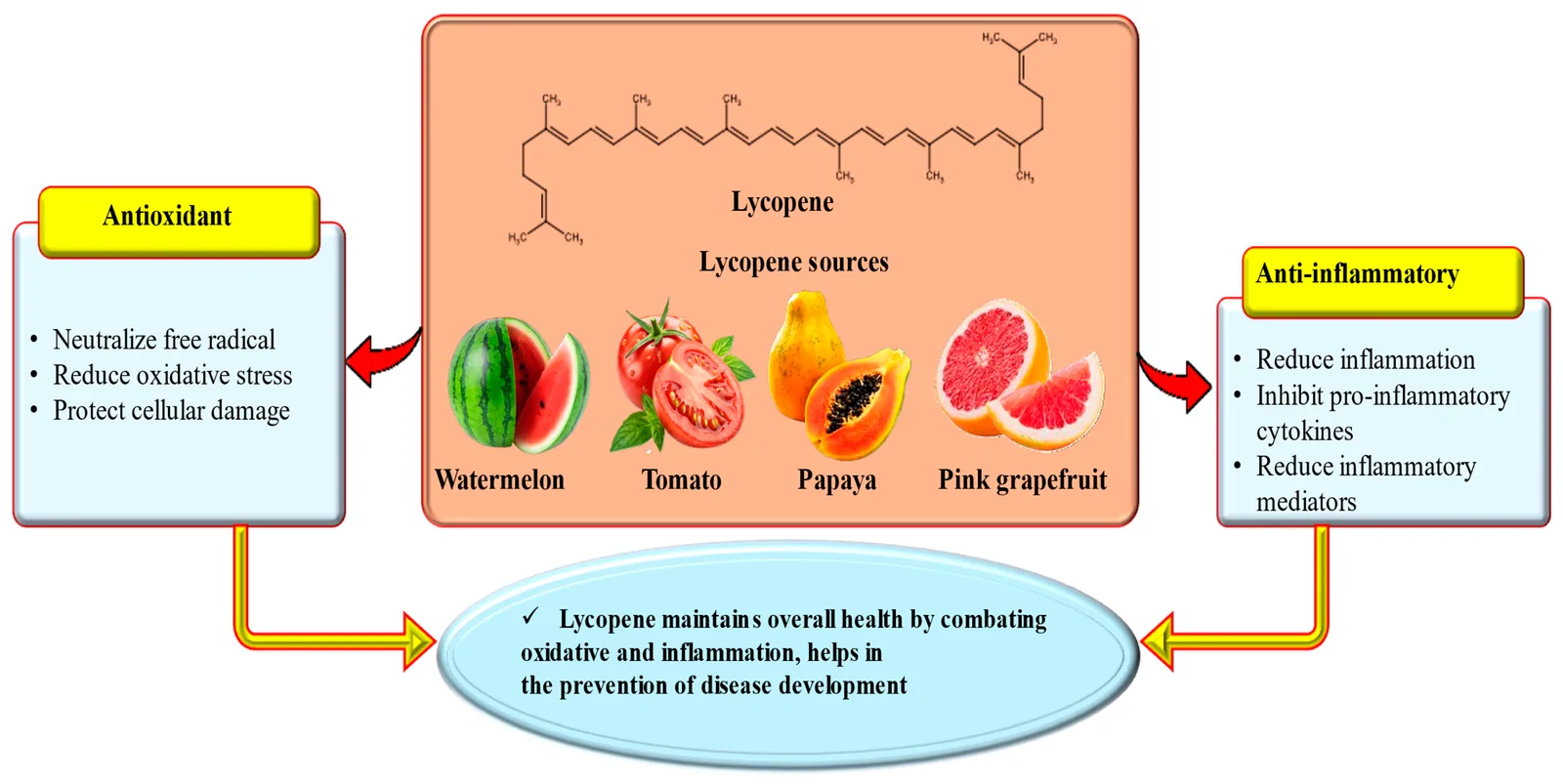
Health-promoting potential of lycopene and its main dietary sources. As an antioxidant and anti-inflammatory, lycopene neutralizes free radicals, reduces oxidative stress, and reduces inflammation and finally protects cellular damage and prevents the development of diseases.

Lycopene is a carotenoid with molecular formula C40H56, and its relative molecular mass is 536.85 g/mol. Moreover, lycopene, as an aliphatic straight-chain hydrocarbon, comprises two unconjugated double bonds as well as 11 conjugated bonds [12]. Lycopene exists in both trans and cis forms, with the cis isomers being more easily absorbed and having higher bioavailability compared to trans-lycopene [13,14]. Lycopene is mainly found in red or orange fruits and vegetables, including Carica papaya, Solanum lycopersicum, Psidium guajava, Punica granatum, and Citrullus lanatus [15]. Additionally, lycopene is also present in asparagus and Petroselinum crispum [16]. Moreover, red fruits as well as vegetables such as tomatoes, apricots, pink guavas, watermelons, and pink grapefruits are imperative sources of lycopene [17] (Figure 1). In addition, the chief sources of lycopene in the diet are tomatoes as well as tomato-based products [18]. Lycopene is a powerful antioxidant and anti-inflammatory with many health benefits, and daily intake varies from country to country. The study focused on the daily intake of carotenoids from the Italian total diet. The Italian total diet provided 14.3 mg of carotenoids per person per day. Lycopene constitutes the largest portion of carotenoids in the Italian diet, with an average intake of 7.4 mg/day, making it the most predominant carotenoid [19]. The average daily intake of lycopene differs by country to country as follows: it is stated to be 1.1 mg in the United Kingdom, 4.9 mg in the Netherlands, 1.6 mg in Spain, 3.8 mg in Australia, and 4.8 mg in France [20]. In the USA, the daily intake is more than 7 mg [21]. Additionally, other studies have reported that the mean lycopene intake among Belgian adults was 0.059 mg/kg body weight per day [22].

## 4. Role of Lycopene in Disease Management Through Modulation of Various Biological Activities

Lycopene has a proven role in managing various pathogenesis primary mechanisms by which this compound exerts its health-promoting or disease-cure potential by reducing oxidative stress and inflammation. Moreover, its vital role in many disease preventions has been described through the regulation of many biochemical parameters and the maintenance of tissue architecture. Here, the role of lycopene in different pathogenesis or health management is discussed as follows:

### 4.1. Antioxidant Activity

When the antioxidant defense system is unable to counteract excess free radicals effectively, a disproportion can occur between oxidants as well as the defense mechanisms. This imbalance may result in various pathological conditions, such as cancer [23,24] and cardiovascular disease [25,26]. Plant products have a confirmed role in disease management through their antioxidant potential [27,28,29]. As an antioxidant lycopene neutralizes free radicals, reduces oxidative stress, and finally protects cellular damage and prevents the development of diseases [Figure 1]. A study was conducted to examine the role of lycopene in lipopolysaccharide (LPS)-induced oxidative stress and inflammatory cascades. Lycopene ameliorated LPS-stimulated oxidative stress through total antioxidant enhancement and HDL-associated PON-1 activity, in addition to down-regulating the expression as well as plasma level of inflammatory mediators [30]. The role of lycopene on oxidative stress-caused cognitive defects was examined. It was demonstrated that lycopene decreased levels of inflammatory cytokines and raised activities of antioxidant enzymes in the d-galactose-treated mice serum [31]. The role of lycopene on mitochondria in cultured rat cortical neurons exposed to Aβ was examined. It was noticed that lycopene reduced Aβ-induced oxidative stress, as demonstrated by the decreased mitochondria-derived superoxide production and intracellular reactive oxygen species production. Moreover, lycopene mitigated Aβ-induced mitochondrial structural damage, ameliorated the opening of mitochondrial permeability transition pores, as well as the subsequent release of cytochrome c [32]. The possible protective impacts of lycopene and rosmarinic acid alone or combined on gentamicin-induced renal cortical oxidative stress, apoptosis and autophagy were estimated. Administration of lycopene and rosmarinic acid reduced elevated serum creatinine, renal malondialdehyde, and blood urea nitrogen induced by gentamicin [33]. Further study reported that lycopene reduced the production of ROS in SK-Hep-1 cells. Lycopene improved liver toxicity by acting as an antioxidant and decreased oxidative damage [34]. Another study based on findings concluded that lycopene prevents RCAN1-mediated apoptosis through reduction in ROS levels as well as by inhibiting Nucling induction, NF-κB activation, and the apoptotic indices increase in neuronal cells [35]. When lycopene is in low doses, it acts as an antioxidant, while when present in high doses, it works as a pro-oxidant [36]. A study was made to examine whether lycopene lessens oxidative injury in bMECs caused by H2O2. The outcomes exhibited that treatment with lycopene decreased H2O2-caused accumulation of intracellular ROS [37].

### 4.2. Anti-Inflammatory Activity of Lycopene

Inflammation is a protective response of the immune system triggered by harmful stimuli, including pathogens, cellular damage, toxins, and radiation [38]. Its main function is to remove harmful agents and promote the initiation of tissue repair and healing processes [39]. Therefore, inflammation acts as an essential defense mechanism that plays a critical role in maintaining overall health [40] and protecting the body from injury and infection. Chronic inflammation is long-term inflammation that damages healthy cells and tissues. Lycopene helps modulate inflammatory signaling pathways and reduce the production of pro-inflammatory mediators (Figure 1). Their bioactive constituents are also associated with antioxidant effects, which further contribute to limiting inflammation-induced cellular damage. Sarcandra glabra and lycopene have shown considerable potential in alleviating histopathological damage caused by LPS, enhancing the oxidative stress response in rats, reducing IL-6 and TNF-α levels, inhibition of activation of MAPK pathways, and the transcription factor NF-κB. Overall, this study suggests that the combination of both offers significant protection against LPS-induced acute lung injury [41]. The study explores the impact of lycopene, a powerful antioxidant and anti-inflammatory carotenoid, on the neuroinflammatory cascade induced by intracerebroventricular Aβ1–42. The findings designate that Aβ1–42-induced mitochondrial dysfunction, along with proinflammatory cytokines and caspase-3 activity in rat brain, was meaningfully reduced by the treatment of lycopene [42]. Another study reported that the number of infiltrating cells increased in the aqueous humor of rats in the Vehicle + LPS group. Compared with the Vehicle + LPS group, pretreatment by lycopene markedly reduced oxidative stress and inflammatory markers. In addition, the inflammatory score was meaningfully decreased following lycopene administration [43]. In the study, lycopene was administered via intragastric pretreatment to assess its effects against β-amyloid-induced inflammation. The outcomes showed that lycopene significantly reduced inflammatory cytokine levels and counteracted the Aβ1–42-induced upregulation of NF-κB p65 and TLR4 at both mRNA and protein levels in the choroid plexus [44].

Overall, the studies discussed above demonstrate that lycopene exerts substantial protective effects against oxidative stress and inflammation-mediated cellular and tissue damage by its antioxidant, anti-inflammatory, reduce ROS production, and inflammatory cytokines. The findings support the therapeutic potential of lycopene in managing oxidative stress as well as inflammation-associated pathogenesis.

### 4.3. Role of Lycopene as Hepatoprotective

The liver performs essential functions in maintaining normal physiological balance, including nutrient metabolism, detoxification, immune system regulation, and the maintenance of carbohydrate, protein, and lipid homeostasis [45,46]. Liver disease and its complications are important contributors to illness and death worldwide. Lycopene plays a role in reducing liver function enzymes and maintaining liver tissue architecture (Table 1). The finding observed the role of lycopene on lipoprotein metabolism in rats subjected to hepatitis induced by D-galactosamine/lipopolysaccharide (D-Gal/LPS). The toxic effects of D-Gal/LPS in the experimental group caused a lessening in the normal levels of lipid metabolism enzymes due to liver damage. Remarkably, there was a substantial decrease in HDL levels, accompanied by an increase in VLDL and LDL levels. However, pretreatment with lycopene was found to restore these altered values to nearly normal levels in the experimental animals [47]. Atrazine (ATR) exposure was found to raise total cytochrome P450 (CYP450) as well as cytochrome b5 levels, while also enhancing CYP450 enzyme activities in liver microsomes. It meaningfully influenced the mRNA expression of six CYP450 genes, with reductions observed in CYP2f2, CYP3a11, and CYP4a31 and increases in CYP1a1, CYP2a4, and CYP3a57. Lycopene, however, helped regulate both the content and activity of CYP450 enzymes and normalized the expression of several genes, including CYP1b1, CYP2e1, CYP2a4, and CYP4A14. Overall, these findings suggest that ATR disrupts hepatic CYP450 function and gene expression, whereas lycopene exerts a protective effect by modulating hepatic activities of CYP450s and transcription in mice [48]. Another study reported that tramadol (TD) induced decreased serum protein, globulin, and albumin, an increase in tissue lipid peroxidation biomarker, and disturbance in the antioxidant homeostasis, as well as raising the serum liver injury biomarkers. In TD-challenged rats, hepatic activities and gene expression of glutathione S-transferase (GST), thioredoxin-1 (Txn-1), superoxide dismutase (SOD), and catalase (CAT) were decreased; these changes were mitigated by lycopene. Additionally, fatty degeneration as well as necrosis was reduced by lycopene [49]. The study checked the role of lycopene against oxidative damage during experimental hepatitis, caused by D-galactosamine (300 mg/kg b.w)/lipopolysaccharide (30 µg/kg b.w). Rats were pretreated by lycopene (10 mg/kg b.w for 6 days) and then D-GalN-/LPS-induced. Study outcomes demonstrated that pretreatment with lycopene meaningfully reversed the high levels of lipid peroxides caused by D-GalN/LPS in the animals. Reduced activities of enzymatic as well as nonenzymatic antioxidant markers were restored by lycopene administration [50]. The study was made to examine the protective role of lycopene against hepatic ischemia/reperfusion injury. Rats were subjected to 45 min of hepatic ischemia followed by a 60 min reperfusion period. Lycopene was given i.p at doses of 2.5 and 5 mg/kg b.w, one hour prior to ischemia. Lycopene administration at doses of 2.5 as well as 5 mg/kg b.w led to partial, dose-dependent improvements in ALT, LDH, AST, and MDA levels, while catalase activity was completely restored in a dose-independent way. These findings indicate that lycopene has a protective effect against liver injury [51]. The intoxication of rats by carbon tetrachloride (CCl4) caused substantial histological hepatic degradation, an increase in the number of apoptotic cells and reactive oxygen species ROS, a reduction in the concentration of reduced GSH, and activities of SOD, CAT, GPx, and GST. CCl4-administered rats, pre-treated with lycopene, reduced cell damage as well as ROS generation. The levels of hepatic integrity markers in rats pre-treated with lycopene were similar to the control group when CCl4 treatment was absent, highlighting the protective effect of lycopene pre-treatment. Similarly, lycopene-pre-treated rats meaningfully increased the antioxidant enzyme levels [52]. The role of lycopene on CCl4-induced hepatic fibrosis was determined. In the CCl_4_ group, the rats were given 4 mL/kg of CCl_4_ (dissolved in olive oil) through injection of intraperitoneal for 12 weeks. Meanwhile, in the lycopene group, the rats were given 4 mL/kg of CCl_4_ and lycopene administered orally (10 mg/kg b.w/d). Study results demonstrated that lycopene meaningfully reduced the liver/body weight ratio and ALT and AST levels. Also, histological analysis confirmed that lycopene decreased collagen expression and improved lobular architecture [53].

The hepatoprotective potential of lycopene pretreatment in paracetamol-induced liver damage was examined. Rats were given lycopene orally (4 mg/kg/day) through gastric lavage for 8 days. On day 8, paracetamol 3 g/kg was administered. Remarkably, it was noticed that 24 h after paracetamol-induced liver damage, lycopene treatment meaningfully decreased serum transaminase (ALT/AST) levels as compared to those in the saline-treated group. Lycopene improved liver recovery following paracetamol-induced liver damage [54]. The study aimed to assess the role of lycopene in liver injury. Lycopene decreased thiobarbituric acid-reactive species levels, improved redox imbalance, and increased antioxidant enzyme levels. This study advocates that lycopene consumption diminishes the effects of liver injury [55]. The role of lycopene on LPS (20 mg/kg intraperitoneally injected)-induced liver injury in mice was investigated. Mice were assigned to sham control, lycopene treatment, and LPS control groups. The mice from the lycopene treatment were treated with lycopene for two weeks. The pretreatment by lycopene decreased levels of IL-6, ALT, AST, and TNF-α, increased activity of SOD, and reduced MDA content in serum. The Nrf2 expression increased, as well as the phosphorylation of NF-κB and ERK1/2 by lycopene [56]. The role of lycopene on endoplasmic reticulum stress in CCl4-caused acute liver injury in mice was determined. It was noticed that lycopene reduced the activity of LDH and inhibited apoptosis. The findings concluded that lycopene reduces liver injury [57]. Lycopene demonstrates substantial hepatoprotective activities in several experimental models of liver injury, through reduction in liver injury markers, improves lipid metabolism, restores antioxidant defenses and decreases oxidative stress, inflammation, and fibrosis. Overall, the evidence advocates that lycopene protects the liver through anti-inflammatory, antioxidant, and anti-fibrotic mechanisms, supporting its potential as a therapeutic agent against liver pathogenesis.

### 4.4. Anti-Diabetic Potential

Natural compounds have shown anti-diabetic potential through the reduction in lower blood sugar levels and oxidative stress, improving insulin secretion and decreasing complications related to diabetes [58,59]. Many plant-derived bioactive compounds, such as flavonoids, polyphenolic acid, and carotenoids, have been described to protect pancreatic β-cells and renal cells and enhance glucose metabolism. In diabetes mellitus, the role of lycopene is demonstrated by various mechanisms (Table 2). The effects of caffeine as well as lycopene on streptozotocin (STZ)-induced diabetes mellitus (DM) in rats were evaluated. Noticeable degeneration and vacuolization were seen in STZ-treated groups. Caffeine and lycopene reduced the pathological changes and decreased the urine glucose and blood levels, while these compounds enhanced serum insulin levels. The finding concluded that lycopene and caffeine showed protective role against DM. The effects of lycopene were noticed to be greatly better than caffeine [60]. The purpose of this study is to evaluate the capacity of lycopene against diabetes-induced oxidative damage in rats. Administration of graded doses of lycopene to diabetic animals reduced the blood glucose concentration after four weeks of treatment. Cortisol and MDA reduced and upregulated activities of endogenous enzymes serum levels in diabetic animals treated by lycopene as compared with diabetic group animals [61]. The effect of lycopene on the metabolism of glycolipid in type 2 diabetes was checked. Diabetic group animals exhibited an increase in fasting blood glucose, liver, glycosylated hemoglobin, HOMA-IR, and lipid in blood, and a decline in plasma insulin compared to the normal control group. It was reported that oral administration of lycopene oil solution (10 or 20 mg/kg b.w) improves the above alterations toward normality. The activities of oxidative enzymes GSH-Px and SOD increased and decreased MDA in pancreatic tissue by lycopene. Furthermore, lycopene also protects against loss of body weight in diabetic rats [62]. The effects of lycopene on diabetes changes in rats were determined. Administration of lycopene reduced serum glucose, TC, TG, AST, and ALT levels, and enhanced serum insulin levels. The results propose that orally administered lycopene shows a potent hypoglycemic effect. The study outcomes advise that lycopene administrated shows a strong hypoglycemic activity in STZ-caused diabetic rats as well as that lycopene might be valuable for the diabetes mellitus management [63]. Treatment of diabetic rats by lycopene caused a decrease in levels of blood urea nitrogen, 24 h urea protein, and creatinine. In the DM-L group, serum lipids such as total triglycerides, cholesterol, and low-density lipoprotein were lowered, while high-density lipoprotein levels increased compared to diabetic rats. Additionally, lycopene administration led to a decrease in malondialdehyde levels and connective tissue growth factor expression, along with increased phosphorylation of Akt/PKB and SOD activity in the renal tissues of diabetic rats [64]. The study findings designated a noteworthy rise in serum glucose, creatinine, urea, and kidney tissue L-MDA levels, accompanied by an upregulation of NF-kB gene expression in rats with induced diabetic nephropathy. Conversely, there was a significant decline in SOD activity and GSH levels in the kidney tissues. However, the introduction of lycopene to the diabetic nephropathy-induced rats resulted in a substantial improvement in all these parameters, bringing them closer to normal ranges [65]. The study was intended to examine the hypoglycemic potential of lycopene in diabetic rats. The results obtained exhibited that lycopene at all doses reduced the blood glucose after four weeks of treatment. The serum insulin level and activity of hepatic glucokinase increased as compared to diabetic control rats [66].

The administration of STZ (45 mg/kg b.w) caused a significant decrease in body weight and an increase in plasma glucose in rat models. The supplementation of lycopene (10 mg/kg b.w) meaningly reduced diabetic plasma glucose level and prevented loss of body weight by lycopene administration. Lycopene administration significantly decreased lipid peroxidation as well as plasma NO levels [67]. A study was made to assess the therapeutic effects of supplementation of lycopene in diabetic rats. The findings exhibited that lycopene reduced the diabetes-associated increase in blood glucose levels. Also, plasma nitric oxide levels and brain tissue glutathione levels were suggestively declined. In brain tissue homogenates, glutathione peroxidase activity was increased following lycopene treatment. Moreover, the oxidative damage as well as low insulin levels linked with diabetes were bettered by the lycopene administration. The outcomes of the study show that lycopene is a nutritional component that improves and/or prevents diabetes complications [68].

### 4.5. Neuroprotective Effect

**Figure 2:**
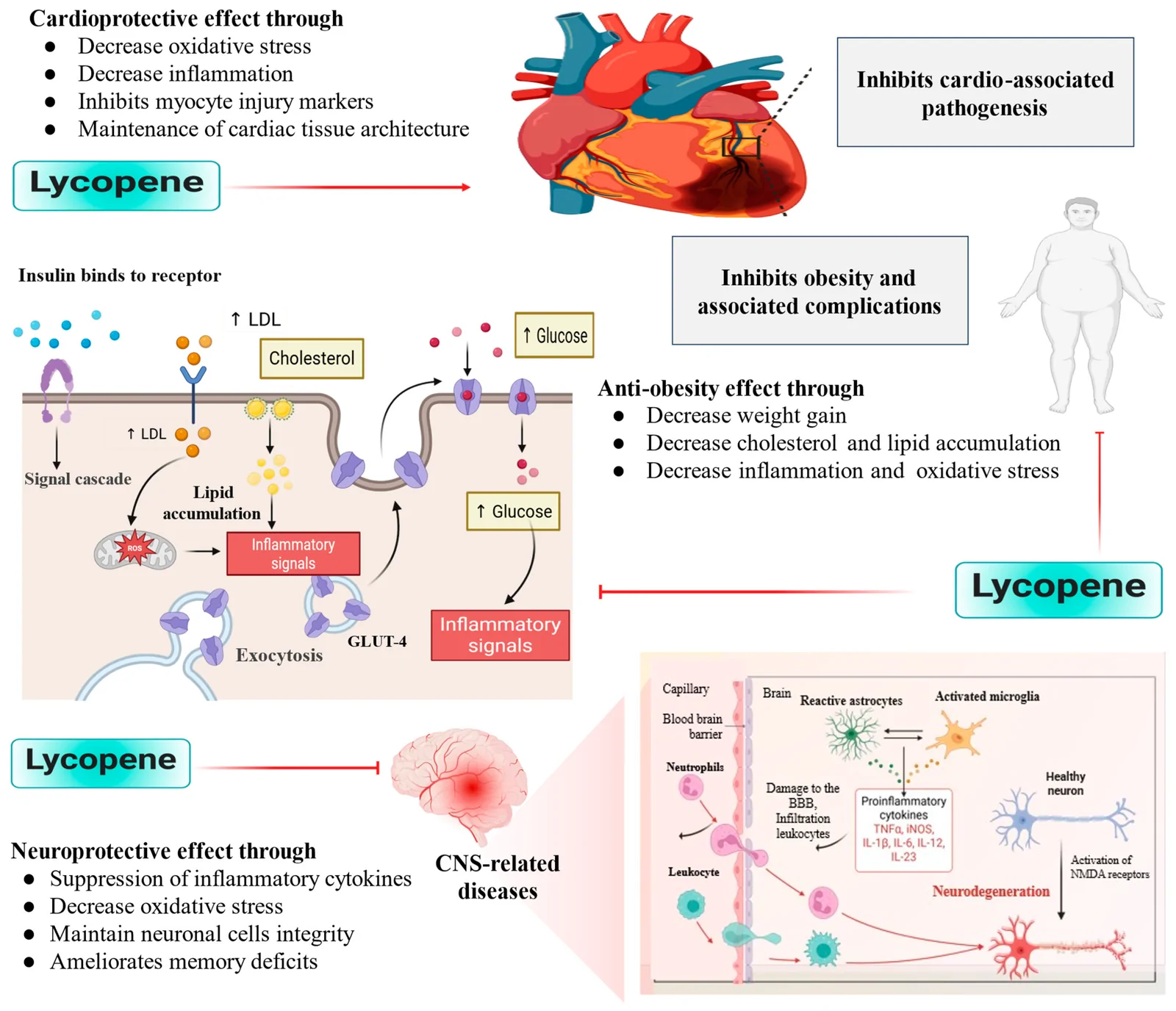
The potential role of lycopene in metabolic, cardiovascular, and neurological disorders. Lycopene exerts cardioprotective and neuroprotective effects by reducing oxidative stress and inflammation and improving tissue integrity. In obesity- and diabetes-related pathways, lycopene modulates lipid accumulation, glucose metabolism, cholesterol, triglycerides, inflammatory signaling, and insulin resistance. The figure was created in https://BioRender.com. Abbreviations: Low-Density Lipoprotein (LDL), Glucose Transporter Type 4 (GLUT-4), Blood–Brain Barrier (BBB), N-Methyl-D-Aspartate (NMDA), Tumor Necrosis Factor Alpha (TNF-α), Inducible Nitric Oxide Synthase (iNOS), Interleukin-1 Beta (IL-1β), Interleukin-6 (IL-6), Interleukin-12 (IL-12), and Interleukin-23 (IL-23).

Lycopene has been revealed to show a neuroprotective role by multiple mechanisms (Table 3). It acts as a powerful antioxidant by reducing oxidative stress in neural tissues, and it maintains neural tissue integrity. Lycopene plays a neuroprotective role through various mechanisms described accordingly (Figure 2). Research proved that lycopene exerts significant neuroprotective effects in both in vivo and in vitro studies. These protective effects are primarily attributed to its strong antioxidant as well as anti-inflammatory properties. Lycopene has also been reported to reduce neuronal damage, inhibit oxidative stress, and improve cognitive function in various experimental models. The study was made to explain the neuroprotective role of lycopene against the β-amyloid induced cognitive impairment as well as mitochondria oxidative damage in rats. β-amyloid was given by intracerebroventricular in rats. Moreover, lycopene (2.5 and 5 mg/kg) was given for 3 weeks. It was reported that chronic lycopene administration caused an improvement in memory retention, reducing neuroinflammation [70].

The study was made to examine the role of lycopene in intracerebroventricular Aβ1–42-induced neuroinflammatory cascade with memory and learning impairment in rats. Aβ1–42 was injected bilaterally and then treated by lycopene or rivastigmine. Lycopene improved Aβ-induced impairments in memory and learning in a dose-dependent way. It also meaningfully alleviated mitochondrial dysfunction and reduced the high levels of proinflammatory cytokines in the rat brain caused by Aβ1–42 [42]. Other study results demonstrated that lycopene reduced hypoxic-ischemic (HI) brain injury *in vivo* as well as oxygen-glucose deprivation (OGD)-induced apoptosis of cortical neurons *in vitro* through the Nrf2/NF-κB signaling pathway [71].

The effect of lycopene on hippocampus in lipopolysaccharide caused by Alzheimer’s disease in rats was inspected. Study results reported that lipopolysaccharide induced an important reduction in brain and body weights, reference memory, as well as neuronal parameters. There were noteworthy histological lesions, demyelination, as well as increased neuroinflammation. Lycopene exhibited neuroprotective effects by suppressing the production of inflammatory cytokines, reducing oxidative stress, and alleviating pathological lesions [72]. Another study was conducted to explore whether lycopene alone or in combination with vitamin E protected against memory impairment in tau transgenic mice expressing the P301L mutation. The results revealed that the combined administration of lycopene and vitamin E produced synergistic antioxidant effects, meaningfully reducing oxidative stress associated with tauopathies [73]. The lycopene administration was found to reduce Aβ accumulation, increase the LRP1 levels, and reduce the RAGE levels in the cerebrospinal fluid or the choroid plexus. In addition, lycopene enhanced the antioxidant enzymatic system, suppressed RAGE/nuclear factor-κB signaling pathway, and produced pro-inflammatory cytokines in the choroid plexus of Alzheimer’s disease rats [74]. The neuroprotective role of lycopene against Parkinson’s disease (PD) in mice was investigated. Lycopene administration protected against the reduction in striatal dopamine as well as its metabolites. It also reduced motor abnormalities and oxidative stress. Moreover, the treatment by lycopene inverted apoptosis. Based on the outcomes, the study demonstrated that lycopene reverses neurochemical defects, apoptosis, physiological abnormalities, and oxidative stress and offers a potential approach in neurodegenerative disease treatment [75]. The neuroprotective activity of lycopene on oxidative stress, as well as neurobehavioral abnormalities, was assessed in a rotenone-induced Parkinson’s disease model in rats. The findings confirmed that co-supplementation with lycopene for 30 days significantly prevented rotenone-induced oxidative stress, improved neurobehavioral deficits, and restored antioxidant status. These outcomes deliver evidence for the beneficial effects of supplementation of lycopene in Parkinson’s disease [76].

The involvement of the nitric oxide mechanism in the protective role of lycopene in 3-nitropropionic acid-caused Huntington’s disease-like symptoms in rats was evaluated. Lycopene administration significantly reduced behavioral, biochemical, and mitochondrial enzyme activity impairments when compared to the group treated with 3-nitropropionic acid. Additionally, pretreatment with L-NAME at 10 mg/kg combined with the sub-effective dose of lycopene (5 mg/kg) meaningfully enhanced its protective effects [77]. Lycopene efficiency in pro-inflammatory, brain-behavior, antioxidant levels, and apoptotic markers, in a rodent model, was studied. Propionic acid (PPA) was given to rats to induce autism-like disorders, and then lycopene (5, 10, and 20 mg/kg/day) was given. The serum and brain MDA levels were reduced by supplements of lycopene, likewise to the brain levels of inflammatory factors. This study exhibited that lycopene might have a therapeutic role to improve the dysfunctions in memory and learning, along with the anti-inflammatory activity [78]. The neuroprotective role of lycopene in kindling epilepsy in mice was checked. Outcomes showed that repeated administration of pentylenetetrazol (PTZ) increased the kindling score, induced oxidative damage, and also impaired mitochondrial enzyme complex activities. Treatment by lycopene, as well as sodium valproate, reduced the kindling score, restored mitochondrial enzyme complex function, and reversed oxidative damage [79].

The neuroprotective role of lycopene in a mouse model was investigated. Lycopene suggestively improved the neurological score and attenuated neuronal apoptosis and oxidative stress caused by global ischemia. Further, the study proved that lycopene exerts a protective role against global ischemic brain injury through its activation of the Nrf2/HO-1 signaling pathway and anti-apoptotic properties [80].

**Table 3:** Neuroprotective effects of lycopene. This table summarizes the study models, doses and key findings as it protects neuronal integrity, improves cognitive and behavioral functions.

Study Model	Dose	Findings	Refs.
β-amyloid induced Alzheimer’s disease in rats	2.5 and 5 mg/kg	° Lycopene protects cognitive dysfunction.	[70]
LPS-induced Alzheimer’s disease in rats	15 mg/kg	° Lycopene showed neuroprotective effects through reducing the pathologic lesions.	[72]
Alzheimer’s disease in rats	10 mg/kg	° Lycopene administration reduced the LRP1 levels, reduced the RAGE levels. ° Administration of lycopene enhanced antioxidant enzymatic system.	[74]
Parkinson’s disease induced by MPTP in mice	5, 10 and 20 mg/kg	° Lycopene administration protected depletion of striatal dopamine and its metabolites.	[75]
Rotenone-induced model of Parkinson’s disease in rats	10 mg/kg	° Lycopene supplementation along with rotenone prevented changes in antioxidants and neurobehavioral deficits.	[76]
3-nitropropionic acid-caused Huntington’s disease-like symptoms in rats	2.5, 5, 10 mg/kg	° Lycopene administration reduced behavioral, biochemical, and mitochondrial enzyme activity.	[77]
Propionic acid-induced autism spectrum disorders in rats	5, 10, 20 mg/kg	° Lycopene reduces learning and memory impairment.	[78]
PTZ-induced kindling seizures in mice	2.5, 5 and 10 mg/kg	° Treatment reduced kindling score.	[79]
Cerebral ischemia–reperfusion in rats	20 mg/kg	° Lycopene improved the neurological score.	[80]

### 4.6. Cardioprotective Effects

Cardiovascular diseases (CVDs) are the main cause of death worldwide [81], accounting for around 10 million deaths annually, a number projected to increase to 23.6 million by 2030 [82]. Lycopene exerts cardioprotective effects by multiple biological mechanisms. Furthermore, lycopene improves lipid metabolism, protects vascular function, and helps prevent the progression of cardiovascular-associated pathogenesis. Lycopene plays a role in cardioprotective effects through various mechanisms, as outlined in (Figure 2 and Table 4). The cardioprotective role of lycopene against isoproterenol (ISP)-induced myocardial infarction was examined in rats. Lycopene is orally given in doses of 0.5, 1.0 and 1.5 mg/kg for 30 days, with simultaneous ISP (85 mg/kg) injected subcutaneous on days 28 as well as 29. It was noticed that lycopene pretreatment reduced ISP-induced cardiac dysfunction. Pretreatment with lycopene also prevented the depletion of antioxidants and myocyte injury marker enzymes. Furthermore, it reduced necrosis, edema, and infiltration of inflammatory cells on histopathological investigation. Based on the findings, it is proposed that lycopene holds cardioprotective activity and might assist as an adjunct in treatment as well as prophylaxis of myocardial infarction [83]. The cardioprotective effects of lycopene against ATR-induced cardiac injury were examined in mice model. Lycopene was given to mice at dose of 5 mg/kg and/or atrazine (ATR) (50 or 200 mg/kg) for 21 days. Lycopene confirmed a protective role by reducing histological damage to the heart, and lycopene supplementation meaningfully mitigated cardiac injury. Overall, it was concluded that lycopene caused chemoprotective effect against ATR-caused cardiac injury through the inflammatory response suppression [84]. It was reported that isoproterenol (ISO)-treated rats exhibited noteworthy changes in heart weights, heart rates, and serum lipid profiles. The study confirmed that lycopene supplementation to ISO rats meaningfully ameliorated lysosomal membrane damage as well as the alterations in lipid profile, cardiac enzymes, and oxidative stress markers [85]. Another study reported that rats administered with ISO exhibited a clear increase in the serum marker enzyme levels as well as tissue oxidative stress markers. Histological features of the heart also designated cardiac myofibril’s structure disruption in ISO-intoxicated rats. Quercetin and lycopene (QL) pretreatment prevented all these adverse effects and reduced the myocardial damage [86]. The effect of lycopene against palmitate-invoked cardiotoxicity was examined. Palmitate (PA) increased excessively in phospholipids, cardiac cholesterol as well as non-esterified fatty acids but decreased triglyceride levels. Furthermore, hydrogen peroxide myeloperoxidase and malondialdehyde increased. Also, serum creatine kinase activities, cardiac gamma-glutamyl transferase, NF-kB, IL-6 and IL-1β, mRNA expression increased. Hyperemia, infiltration of inflammatory cells and cardiac interstitium congestion were caused by PA. However, lycopene treatment upturned the features of histological changes and cardiotoxicity caused by PA [87]. The study findings indicate that in moderately overweight, middle-aged individuals, increased lycopene intake leads to alterations in HDL (2 and 3), which increase their antiatherogenic properties. Overall, the outcomes support the cardioprotective effects of higher lycopene consumption [88]. The study was conducted to assess whether diclofenac sodium (DFS) aggravates the cardiotoxic effects of tulathromycin and to determine the protective potential of lycopene against cardiac damage induced by both agents. Compared with the control group, administration of tulathromycin or DFS, either individually or in combination, caused a noteworthy rise in serum biomarkers associated with cardiac injury. Moreover, there was a noticeable rise in tissue nitric oxide and malondialdehyde levels, alongside a notable reduction in reduced glutathione content and the activity of key antioxidant enzymes. Histopathological and immunohistochemical analyses further revealed elevated pathological scores. These changes were more severe in the group treated by both tulathromycin and DFS compared with mice receiving either drug alone. Remarkably, co-administration of lycopene with tulathromycin and/or DFS suggestively diminished these adverse effects [89]. A study reported that atrazine (ATR) led to reduced creative kinase (CK) activity and increased histological changes. Additionally, a substantial change in Na+, K+ and Ca2+ content and the downregulation of Ca2+-ATPase and Na+-K+-ATPase activities and the mRNA expression of their subunits were detected in ATR-exposed mice. Remarkably, supplementary lycopene meaningfully protected the heart against ATR-induced injury [90]. Another study was made to explore the role of lycopene on adriamycin (ADR)-caused heart and kidney toxicity in rats. Study based on findings concluded that adriamycin (ADR) treatment noticeably impaired cardiac as well as renal function, and treatment with lycopene prevents this toxicity in rats [91].

Overall, based on the above findings, lycopene plays a significant role in cardiovascular protection by regulating oxidative stress and inflammation and reducing pathological changes.

**Table 4:** Cardioprotective effects of lycopene. This table summarizes studies evaluating the cardioprotective effects of lycopene, including study models employed, the doses administered, and the major findings.

Study Model	Dose	Findings	Refs.
ISP-induced myocardial infarction (MI) in rats	0.5, 1.0 and 1.5 mg/kg	° Pretreatment with lycopene attenuated cardiac dysfunction. ° Pretreatment with lycopene prevented the depletion of antioxidants.	[83]
Atrazine-induced cardiac inflammation in mice	5 mg/kg	° Lycopene alleviates cardiac damage.	[84]
ISO-induced oxidative stress and heart lysosomal damage in rats	4 mg/kg	° Lycopene supplementation ameliorated lysosomal membrane damage and alterations in cardiac enzymes.	[85]
Isoproterenol cardiotoxicity in rats	QL (80 mg/kg QN and 3 mg/kg LY)	° QL pretreatment reduced the myocardial damage. ° Administration of lycopene enhanced antioxidant enzymatic system.	[86]
Palmitate-mediated myocardial inflammation in rats	24 and 48 mg/kg	° Lycopene supplementation decreased myocardial inflammation, dyslipidemia, cardiac lipotoxicity, and oxidative stress.	[87]
Tulathromycin and diclofenac sodium-induced cardiotoxicity in mice	10 mg/kg	° Lycopene attenuates cardiotoxicity.	[89]
Atrazine-induced cardiotoxicity mice	5 mg/kg	° Supplementary lycopene combated cardiotoxicity via the regulation of ATPase activity.	[90]
Adriamycin-induced cardiotoxicity rats	4 mg/kg	° ADR treatment noticeably impaired cardiac function and lycopene treatment prevents this toxicity in rats.	[91]

Overall, the studies described above reveal that lycopene shows significant therapeutic potential as hepatoprotective, cardioprotective, neuroprotective, and anti-diabetic effects. These effects are mainly mediated through the regulation of oxidative stress and inflammation, modulation of multiple biological activities, and maintenance of tissue architecture. Collectively, these findings support the potential role of lycopene in the management and prevention of disease conditions.

### 4.7. Anti-Cancer Effects

Cancer is a multifactorial disease, and mortality rates from cancer remain high worldwide. Current treatment modules are costly and often result in numerous side effects. So, safe, less expensive treatment options are imperative to address the limitations of existing treatments.

**Figure 3:**
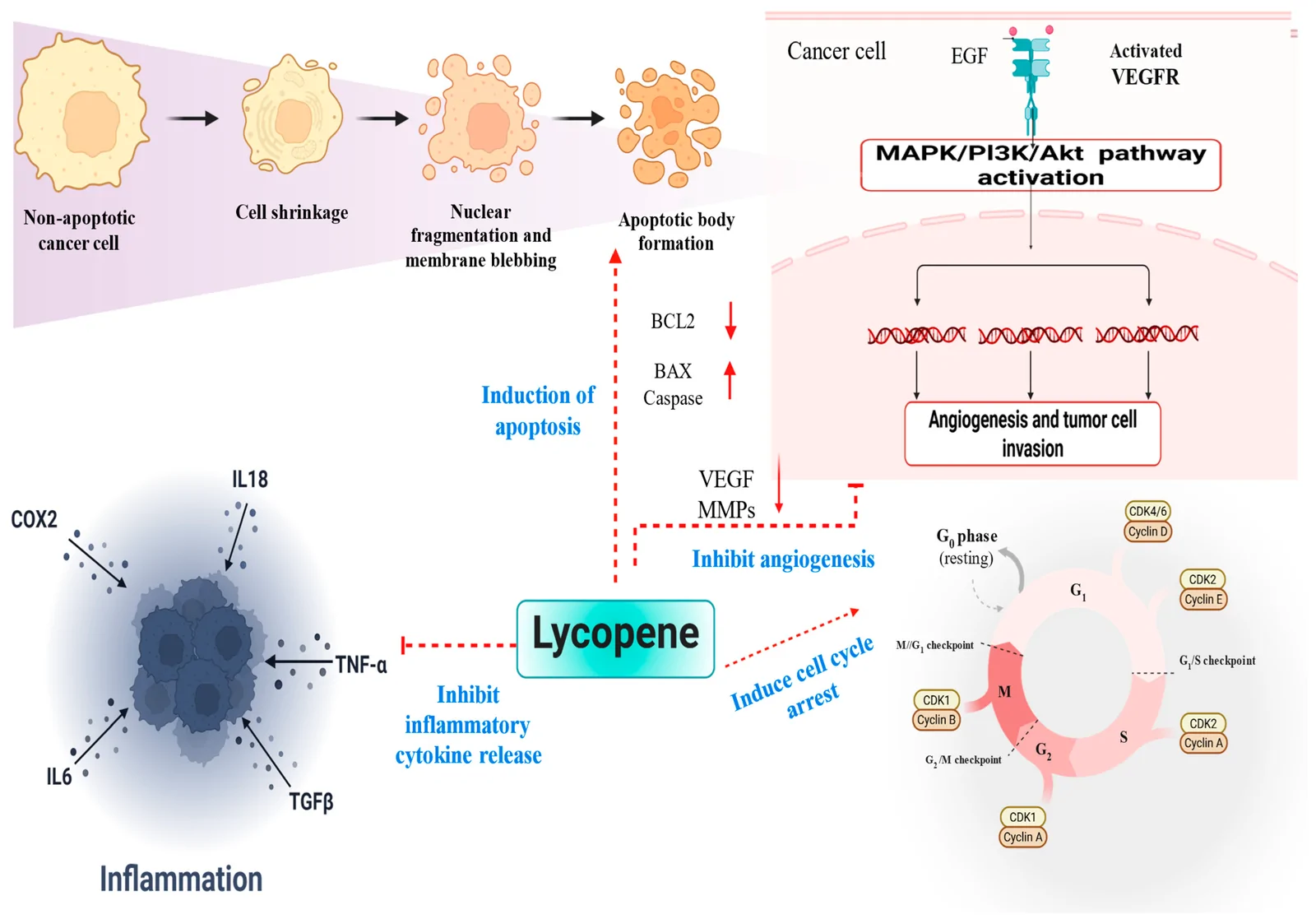
The anticancer mechanisms of lycopene. Lycopene promotes apoptosis and suppresses tumor progression and metastasis by inhibiting angiogenesis-related factors. It also induces cell cycle arrest, thereby halting progression at key checkpoints. Collectively, these actions highlight lycopene’s role in inhibiting cancer development. The figure was created in https://BioRender.com. Abbreviations: Cyclin-Dependent Kinase 4/6 (CDK4/6), Cyclin-Dependent Kinase 1 (CDK1), Cyclin-Dependent Kinase 2 (CDK2), Epidermal Growth Factor Receptor (EGFR), Mitogen-Activated Protein Kinase (MAPK), Phosphoinositide 3-Kinase (PI3K), Protein Kinase B (Akt), Vascular Endothelial Growth Factor Receptor (VEGFR), B-cell Lymphoma 2 (BCL2), BCL2-associated X protein (BAX), Vascular Endothelial Growth Factor (VEGF), Matrix Metalloproteinases (MMPs), Cyclooxygenase-2 (COX-2), Tumor Necrosis Factor Alpha (TNF-α), Inducible Nitric Oxide Synthase (iNOS), Interleukin-1 Beta (IL-1β), Interleukin-6 (IL-6), and Interleukin-18 (IL-8).

In this respect, naturally occurring compounds and their bioactive constituents have shown considerable potential in cancer management due to their ability to control numerous cellular and molecular pathways involved in tumor development as well as progression [92,93,94,95]. As a result, lycopene is increasingly being examined as a promising agent for cancer prevention. This is discussed below and outlined in Table 5 and Figure 3.

i. Inflammation

In the nineteenth century, German physician Rudolf Virchow first observed the connection between inflammation and tumors [96]. Chronic inflammation processes are a key innate immune response to disrupted tissue homeostasis and play a crucial role at various stages of tumor development, such as initiation, promotion, malignant transformation, invasion, and metastasis [97]. It is now widely recognized that the presence of inflammatory cells precedes tumor development [98,99]. Since all tumors show signs of inflammatory infiltration, chronic inflammation is recognized as a hallmark of cancer [100,101].

Natural compounds contribute meaningfully to cancer prevention, and they help in the decrease in the production of pro-inflammatory cytokines and downregulation of the expression of genes associated with inflammation. A study investigated how lycopene affects the level of inflammatory factors in prostate cancer cells. Prostate cancer cells, DU145 LNCaP, and PC3 were used in this study. In all three cell lines, lycopene treatment caused a decrease in cell viability. It was examined how lycopene affects the level of inflammatory factors in prostate cancer cells. Prostate cancer cells were treated by lycopene, followed by assessment of the expression of IL1, IL6, IL8, and TNF-α. The data exhibited that prostate cancer cells confirmed important upregulation of all these inflammatory factors; lycopene treatment reduced this upregulation [102]. Anti-inflammatory potential of lycopene in human colorectal cancer cells (SW480) was checked. Lipopolysaccharide (LPS)-stimulated colorectal cancer cells were treated by lycopene (0, 10, 20, and 30 µM). In cells treated by lycopene as well as LPS, the mRNA expression of TNF-α, IL-6, COX-2, iNOS, and L-1β was reduced meaningfully. The concentrations of NO, PGE2, and protein expressions of JNK and NF-κB were reduced according to the lycopene concentration [103]. The study examined whether lycopene suppresses HFD-promoted hepatocellular carcinoma progression and if BCO2 expression is significant using BCO2-knockout (BCO2-KO) as well as wild-type male mice. It was noticed that the chemopreventive effects of lycopene in wild-type mice were associated with hepatic proinflammatory signaling and a decrease in inflammatory foci. It is also reported that supplementation of lycopene in BCO2-KO mice suppressed oncogenic signals [104].

ii. Angiogenesis

Tumors meet their demands for oxygen and nutrients by creating new blood vessels to support various metabolic processes [105]. Tumor angiogenesis was originally characterized by the proliferation and migration of vascular endothelial cells, which utilize existing capillaries or veins to form a new vascular network [106]. The efficiency of lycopene against the growth of prostate cancer in vivo was evaluated. Athymic nude mice were implanted subcutaneously by androgen-independent prostate carcinoma PC-3 cells. They were supplemented by a low or a high dose of lycopene and a single dose of β-carotene. At the end of the experiment, both β-carotene and lycopene inhibited tumor growth, as demonstrated by the reduction in tumor weight and tumor volume. β-carotene and high-dosage lycopene suggestively increased the levels of insulin-like growth factor-binding protein-3 and reduced the expression of proliferating cell nuclear antigen in tumor tissues and high-dosage lycopene reduced the VEGF levels in plasma. However, supplementation of β-carotene increased the VEGF levels [107]. A study was performed as nude mice were orally supplemented twice per week for twelve weeks with a low or high dose of lycopene or with *β*-carotene. Two weeks after the start of supplementation, animals were injected once with human hepatoma SK-Hep-1 cells. The plasma levels of matrix metalloproteinase-2 and vascular endothelial growth factor (VEGF) increased slowly in tumor-injected mice following tumor injection, while being noticeably lowered by lycopene or *β*-carotene supplementation. High-lycopene supplementation also decreased the level of VEGF and VEGF in lung tissues [108].

iii. Apoptosis

Apoptosis is a stringently organized process, controlled by a sequence of signal transduction cascades and cellular proteins [109]. Failure to initiate complete apoptosis in an unhealthy cell population can cause cells to grow uncontrollably, leading to the development of cancer [110]. Lycopene plays a substantial role in cancer management, chiefly by its ability to induce apoptosis in malignant cells. It affects key regulatory pathways by modulating anti-apoptotic and pro-apoptotic proteins, in that way promoting the elimination of cancerous cells. The role of lycopene on apoptosis of human prostate cancer (PCa) cells was examined. Flow cytometer investigation demonstrated that lycopene was promoted up to a two-fold increase in apoptotic cells in PCa cells as compared to the control group [111]. The study was made to examine the mechanisms of lycopene-induced apoptosis in gastric cancer cells. The human gastric cancer cell line AGS was used in this study. The findings reveal that lycopene significantly decreases cell viability by increasing the Bax/Bcl-2 ratio and promoting DNA fragmentation. The results presented that lycopene induces apoptosis through suppressing β-catenin-c-myc/cyclin D1 axis and reducing ROS levels. Also, results suggest that lycopene induces apoptosis through disrupting the nuclear translocation of β-catenin as well as downregulation of the expression of key genes involved in cell survival [112]. The anti-cancer mechanism of lycopene was investigated by measuring the expression levels of inhibitors of apoptosis in human pancreatic cancer cells, PANC-1. The outcomes of the study demonstrated that lycopene induces the apoptosis of pancreatic cancer cells by suppressing the expression of survivin, cIAP1, and cIAP2 [113].

The role of lycopene at physiologically achievable concentrations on cellular proliferation, apoptosis, as well as necrosis in human prostate cancer cells (LNCaPs) was checked. Study finding confirmed that increasing concentrations of lycopene reduced MMP, triggered the release of cytochrome c from the mitochondria, and enhanced annexin V binding important apoptosis indicators [114].

iv. Cell cycle

Lycopene plays a substantial role in inhibition of cancer cell growth by causing cell cycle arrest. A study based on prostate cancer was performed to check the activity of lycopene on the cell cycle. In PCa cells, lycopene treatment decreased the cells in G2/M phase and increased cells in the G0/G1 phase [111]. 

The study was made to inspect whether lycopene reduces the mitogenic effects of IGF-I in MCF7 mammary cancer cells. Lycopene treatment suggestively reduced IGF-I-induced tyrosine phosphorylation of insulin receptor substrate-1 and reduced the binding activity of the AP-1 transcription complex. The inhibitory role of lycopene on IGF signaling was linked with IGF-stimulated cell cycle progression suppression of serum-starved, synchronized cells. Furthermore, in cells synchronized through mimosine treatment, lycopene cell cycle progression was delayed after release from the mimosine block [115]. The study examined the impact of lycopene on cell cycle and apoptosis in human prostate cancer cells (LCNaPs). Lycopene at a concentration of 1 µM was found to reduce cell growth by 31% when compared to placebo. At a concentration of 5 µM, lycopene increased the proportion of cells in the G2/M phase from 13% to 28%, while reducing the percentage of cells in the S phase from 45% to 29% [116]. Another study was made to determine the role of lycopene in the cell cycle in human cancer cell lines. It was reported that lycopene induced cell cycle arrest and decreased cell viability compared to control groups [117]. Research indicates that both lycopene and apo-12′-lycopenal play a role in reducing human prostate cancer cell proliferation at supra-physiological concentrations through inhibiting the progression of the cell cycle [118]. Using triple-negative MDA-MB-468, ER/PR positive MCF-7, and HER2-positive SK-BR-3 cell lines, the molecular and cellular mechanism of the anticancer effects of lycopene were examined. The treatment of lycopene for 168 successive hours revealed time-dependent as well as dose-dependent anti-proliferative effects against these cell lines through arresting the cell cycle at the G0/G1 phase. Lycopene induced strong as well as sustained ERK1/2 activation, with associated p21 upregulation and cyclin D1 suppression [119]. The role of lycopene in regulating cell viability, apoptosis, as well as cell cycle progression in prostate cancer cells was examined. Lycopene treatment reduced cell viability across all cancer cell lines, increased the proportion of cells in the S and G2/M phases, and decreased the % of cells in the G0/G1 phase. Flow cytometry examination of cell cycle demonstrated that lycopene indorsed cell cycle arrest in G0/G1 phase after 48 as well as 96 h of treatment in a primary cancer cell line. Also, induction of apoptosis by lycopene in prostate cancer cells was noticed [120]. Another study based on breast cancer cell lines reported that beta-carotene and lycopene increase apoptosis and arrest the cell cycle in different phases [121]. To check whether the decrease in cyclin D1 level is the main mechanism for lycopene as well as atRA inhibitory action on IGF-I-induced cell cycle progression. The endometrial (ECC-1) and human breast (MCF-7) cancer cells were synchronized in G0/G1 phase through serum deprivation followed by stimulation with IGF-I. Treatment of cells with lycopene as well as atRA reduced retinoblastoma protein phosphorylation and inhibited IGF-I-stimulated cell cycle progression from G1 to S phase [122].

v. PI3K/AKT pathway

Natural compounds and their ingredients show potential as anticancer agents through their capability to inhibit the PI3K/Akt pathway, an important pathway altered in many cancers. The inhibitory effects of lycopene on the Akt signaling pathway in human colon cancer cells were investigated. Lycopene treatment suppressed Akt activation as well as non-phosphorylated β-catenin protein level [123]. The combined impact of lycopene and eicosapentaenoic acid (EPA) on the growth of human colon cancer cells (HT-29 cells) was explored. The results showed that even at low concentrations, lycopene and EPA worked synergistically to inhibit cancer cell proliferation. This inhibitory effect was associated with the downregulation of the phosphatidylinositol 3-kinase/Akt signaling pathway [124]. The anti-cancer role of lycopene on the progression of oral cancer (OC) was investigated. Lycopene inhibited OC cell proliferation, migration, apoptosis and invasion in a dose-dependent manner. To clarify the causal mechanism the regulatory role of lycopene on OC, the EMT markers and the vital proteins associated with the PI3K/AKT/m-TOR signaling pathway were examined. The treatment by lycopene treatment suggestively and dose-dependently reduced the expression ratios of p-AKT/AKT, p-PI3K/PI3K, as well as p-m-TOR/m-TOR, but ratio of E-cadherin/N-cadherin in OC cells increased [125].

Overall, the evidence presented above indicates that lycopene shows important anticancer potential through multiple mechanisms, including suppression of inflammation and angiogenesis, induction of apoptosis, regulation of cell cycle progression and PI3K/AKT pathway. Although lycopene has confirmed hopeful anticancer mechanisms in cell line as well as animal studies, these findings remain mainly preclinical. Current evidence from human studies is still inadequate, and modulation of molecular pathways does not certainly confirm clinical anticancer efficacy in humans. Thus, further well-designed clinical studies are essential to establish the therapeutic significance of lycopene in cancer prevention and treatment.

**Table 5:** Anti-cancer effects of lycopene. This table summarizes the reported anti-cancer effects of lycopene, highlighting the major cell signaling pathways involved, the types of malignancies studied, the experimental models used, and the key findings as ability to modulate signaling pathways related to apoptosis, cell cycle regulation, angiogenesis, and inflammation across various cancer types.

Action On	Cancer Types	Cell Lines/Animal Model	Outcome	Refs.
Inflammation	Prostate	LNCaP, PC3 and DU145	° Lycopene lowered the inflammatory factors expression.	[102]
	Colorectal	SW480	° Lycopene decreases the expression of inflammatory marker in cancer cells.	[103]
	Liver	Mice model	° Lycopene reduced hepatic pro-inflammatory signaling and inflammatory foci.	[104]
Angiogenesis	Prostate	Prostate tumor cells xenografted in nude mice	° High-dosage lycopene supplementation decreased VEGF levels.	[107]
Apoptosis	Prostate	PCa	° Lycopene increased apoptotic cells.	[111]
	Gastric	AGS	° Lycopene induces apoptosis.	[112]
	Pancreatic	PANC-1	° Lycopene induces apoptosis.	[113]
	Prostate	LNCaP	° Lycopene reduced mitochondrial transmembrane potential.	[114]
Cell cycle	Prostate	PCa cells	° Lycopene increases in cells in G0/G1 phase and decreases in G2/M phase.	[111]
	Prostate	LCNaP	° Lycopene increased the number of cells in the G2/M phase of the cell cycle and decreased S-phase cells.	[116]
PI3K/Akt/mTOR signaling pathway	Colon	HT-29	° Lycopene treatment suppressed Akt activation; ° Lycopene and EPA synergistically block downstream mTOR activation.	[123]
	Oral	CAL-27, SCC-9	° Lycopene inhibited epithelial–mesenchymal transition and induced apoptosis.	[125]
PPARγ- LXRα-ABCA1 pathway	Prostate	LNCaP	° Proliferation of prostate tumor cells was inhibited by lycopene via activation of PPARγ-LXRα-ABCA1 pathway.	[126]
Wnt-TCF signaling pathway	Breast	MCF-7 and MDA-MB-231	° Lycopene synergistically increases quinacrine potential and inhibits Wnt-TCF signaling.	[127]
Protein kinase B and MAP kinase signaling pathway	Head and neck	FaDu and Cal27	° Lycopene was found to suppress protein kinase B and MAPK signaling pathways.	[128]

### 4.8. Anti-Obesity Effect

Obesity is characterized by excessive accumulation of body fat and may result from multiple contributing factors, including genetic predisposition, environmental influences, dietary patterns, lifestyle habits, and various clinical conditions [129,130]. Obesity is a consequence of an energy imbalance where caloric intake exceeds caloric expenditure. When this imbalance persists over an extended period, it can lead to metabolic disorders [131]. Several studies have confirmed that dietary bioactive compounds exert anti-inflammatory and antioxidant effects by enhancing thermogenesis as well as energy expenditure while reducing oxidative stress, in that way promoting weight loss and helping to alleviate metabolic disorders [132,133]. The consumption of natural products has been demonstrated to be a promising and safe approach for preventing obesity or acting as anti-obesity potential. The role of lycopene in high fat diet (HFD)-caused obesity as well as metabolic disturbances in rats was checked. Rats fed on HFD and lycopene were orally given 25 and 50 mg/kg or simvastatin (10 mg/kg). Study results reported that long-term consumption of a high fat diet (HFD) meaningfully increased weight gain, cholesterol, liver weight, and Apo-B and LDL-c levels, while decreasing HDL-c levels. Furthermore, high blood glucose and insulin levels, accompanied by low PPAR-γ, were noted in the HFD group. As compared to the model group, lycopene was capable of reversing HFD-mediated changes [134]. The study outcomes exhibited that increased weight gain, liver weight, and triglyceride and cholesterol levels were noticed in long-term consumption of HFD rats. Inflammatory markers and lipid peroxidation levels were higher in HFD rats. Lycopene supplementation effectively reversed HFD-induced changes. Furthermore, liver and white adipose tissue histopathological findings displayed that lycopene treatment restored the injured tissue. Thus, lycopene showed a therapeutic role in managing obesity and its related pathogenesis [135]. It was reported that lycopene remarkably suppressed HFFD-increased body weight gain in mice. Lycopene blocked lipid accumulation in adipose tissue by increasing the expression of lipidolysis-related genes and decreasing the expression of lipogenesis genes. This study demonstrates that the supplementation of lycopene might be a nutritional preventive approach to battle obesity [136].

### 4.9. The Effect of Lycopene on Colon Health

**Figure 4:**
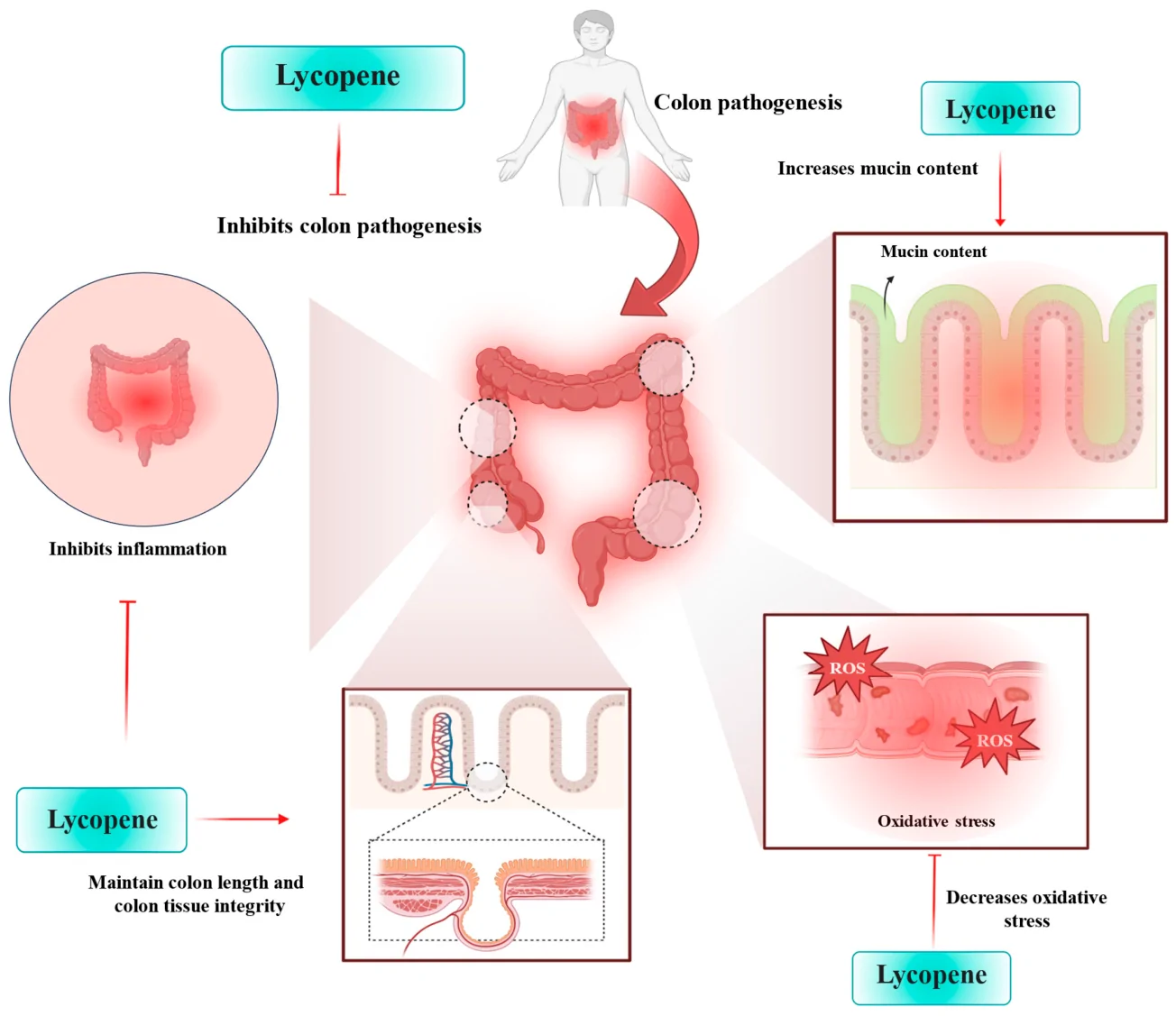
The protective effects of lycopene on colon pathogenesis. It enhances mucin production, contributing to improved mucosal barrier function. Additionally, lycopene helps maintain normal colon morphology, reduce oxidative stress, reactive oxygen species (ROS) and inflammation, collectively mitigating pathological changes. The figure was created in https://BioRender.com.

Lycopene exerts anti-colitis role mainly by reducing oxidative stress and suppressing inflammatory stress, which is commonly activated in intestinal inflammation. It also helps maintain intestinal barrier integrity by reducing epithelial damage in the colon. Lycopene shows anti-colitis effects through different mechanisms (Table 6 and Figure 4). Experimental studies further indicate that lycopene decreases the production of pro-inflammatory cytokines, thereby alleviating tissue injury and improving overall colon health. Yunshuang Yue et al., 2024, reported that lycopene reduced dextran sulfate sodium (DSS)-induced colitis and reduced the proportion of *E. coli* [137]. Lycopene ameliorated DSS-induced colitis by inhibiting *E. coli* adhesion and improving epithelial barrier functions [137]. A study was made to evaluate changes in colonic mucosa in induced ulcerative colitis (UC) as well as the ameliorating effects of lycopene. It was noticed that the UC group exhibited surface epithelium loss with destroyed crypts and congested blood vessels with substantial cellular infiltration. Noteworthy reduction in mean area percentage of ZO-1 expression and goblet cell numbers was detected. Ultrastructural alterations were consistent with the light microscopic findings, which demonstrated destructive changes in the columnar and goblet cells. The histological, ultrastructural, and immunohistochemical observations confirmed the ameliorative effect of lycopene against the damage induced by ulcerative colitis [138]. It was noticed that an increase in L-Malondialdehyde with noticeable reduction in reduced glutathione level, as well as catalase activity, was detected in the colon tissue of UC-induced rats. Moreover, a substantial up-regulation of NF-κB, caspase-3, and TGF-β1 and down-regulation of IL-10 and Bcl-2 gene expression levels were detected in the colon of UC-induced rats. However, an important reduction in colon tissue L-MDA and down-regulation of NF-κB, TGF-β1, caspase-3, in addition to a noticeable increase in CAT activity and GSH concentration, and up-regulation of Bcl-2 and IL-10 were detected after lycopene treatment [139]. Another study reported that, as compared to the dextran sulfate sodium (DSS) group, the lycopene group inhibited DSS-induced weight loss, increased the colon length, decreased the disease activity index score, and improved inflammation in the colon. It also increased the levels of antioxidant enzymes in the colon and reduced myeloperoxidase, inflammatory cytokine, and malondialdehyde levels [140]. The experimentation was made to examine the role of lycopene in colitis in rats. It was reported that lycopene treatments decreased DNA fragmentation, MDA, total sialic acid levels, whereas SOD activity, total antioxidant status, ceruloplasmin and iron levels increased. The histopathological assessment also verified the results. Treatment by lycopene bettered the pathological and biochemical changes caused by colitis [141].

### 4.10. Renoprotective Effect

**Figure 5:**
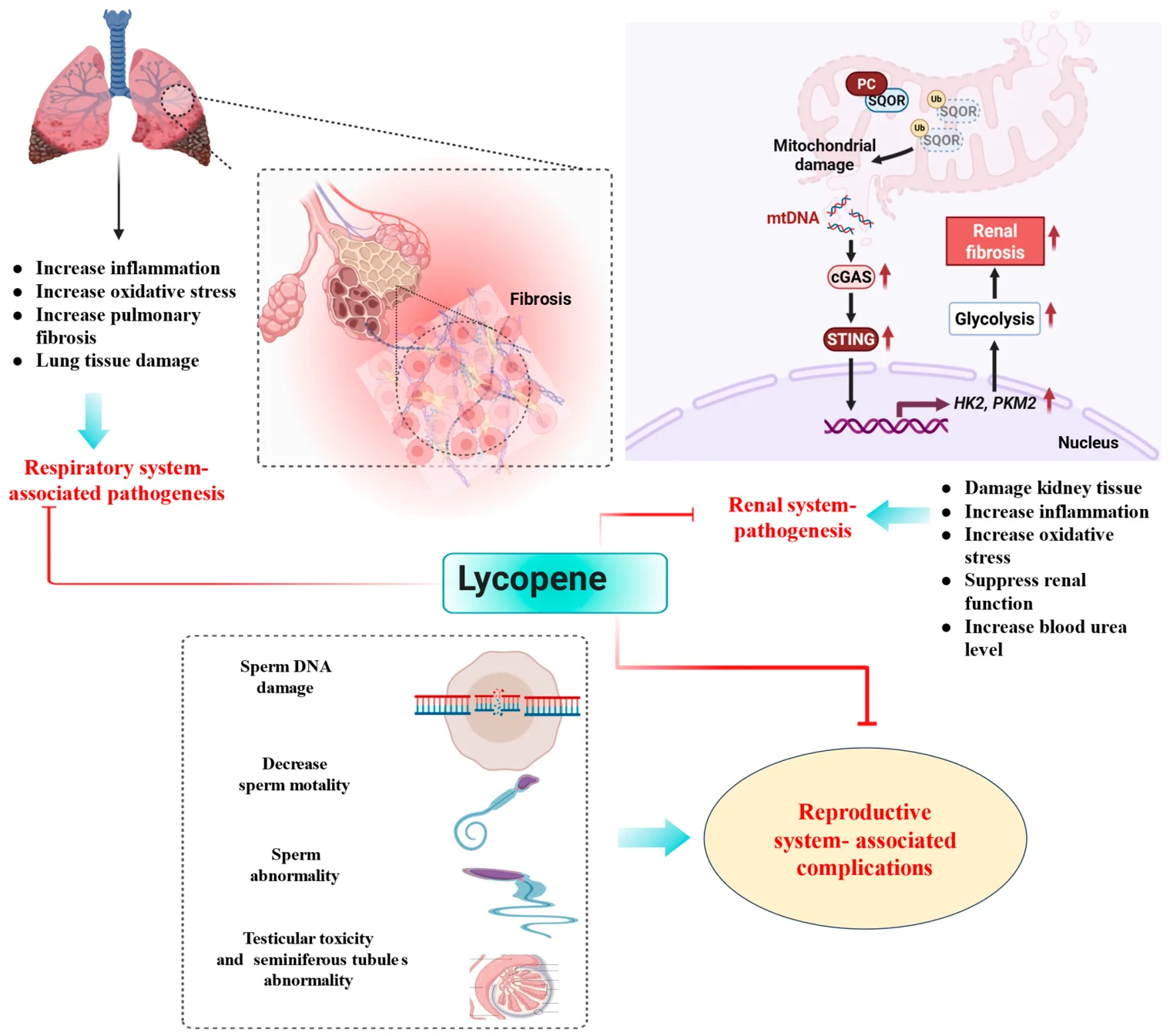
The lycopene role against respiratory, renal, and reproductive system-associated pathogenesis. Lycopene manages pathogenesis mainly through the reduction in inflammation, oxidative stress, and tissue damage. Overall, lycopene attenuates multi-organ complications. The figure was created in https://BioRender.com. Abbreviation: Ub: Ubiquitin, PC: Pyruvate Carboxylase, SQOR: Sulfide Quinone Oxidoreductase, cGAS: Cyclic GMP–AMP Synthase, HK2: Hexokinase 2, PKM2: Pyruvate Kinase M2, mtDNA: Mitochondrial DNA.

The role of lycopene as renoprotective was confirmed through different mechanisms as in Figure 5. A study finding demonstrated that lycopene noticeably ameliorated crystal deposition, restored renal function, and suppressed kidney injury through decreasing fibrosis, oxidative stress, inflammation apoptosis and pyroptosis in the rats. In cell models, pretreatment of lycopene reversed ROS increase, intracellular lactate dehydrogenase release, apoptotic damage, pyroptosis, cytotoxicity, and extracellular matrix deposition. Based on findings, it was concluded that lycopene ameliorates oxalate-caused renal tubular epithelial cell injury [142]. Another study based on results concluded that lycopene supplementation is efficient for renal antioxidant enzymes, expression of ACE gene as well as ACE serum level in hyperlipidaemic rats [143].

Rats fed a high fat diet (HFD) were assessed to study the effects of lycopene against metabolic syndrome as well as renal injury. As compared with the control group, HFD-fed rats revealed a substantial increase in food intake, body weight, serum glucose, creatinine, uric acid, and lipid peroxidation. In addition, kidney tissues from HFD-fed rats exhibited Nrf2 mRNA expression downregulation and a noticeable reduction in antioxidant enzyme activities. Treatment by lycopene efficiently reversed these HFD-induced alterations and improved both metabolic and renal parameters [144]. The study aimed to assess the histopathological and biochemical parameters to examine the role of lycopene in kidney damage induced by rifampicin and isoniazid in rats. Lycopene treatment evidently attenuated these histopathological as well as biochemical alterations. These outcomes advise that lycopene may offer beneficial role against nephrotoxicity linked with isoniazid and rifampicin administration [145].

### 4.11. Role of Lycopene in Respiratory System-Associated Pathogenesis

Respiratory system-associated pathogenesis is a major cause of death globally. The existing modes of treatment are expensive and can also cause adverse effects. The natural compounds have confirmed an important role in prevention of lung pathogenesis. Among these compounds, lycopene has attracted substantial consideration due to its potent antioxidant and anti-inflammatory properties. Lycopene exerts effects against respiratory system-associated pathogenesis and prevention of cellular damage. Lycopene has been reported to improve pulmonary function in various experimental models of lung disease. The role of lycopene in respiratory system-associated pathogenesis, mediated through different molecular and cellular mechanisms, is summarized in Table 6 and Figure 5.

A study based on ovalbumin (OV)-induced murine asthma model was made to check whether lycopene controls inflammatory mediators. Lycopene administration improved airway hyperresponsiveness to inhaled methacholine. It was reported that lycopene administration resulted in a noteworthy inhibition of inflammatory immunocyte infiltration. Furthermore, lycopene reduced the IL-4 expression and increased the levels of GATA-3 mRNA in the OVA-challenged [146]. The matrine as well as lycopene combination treatment deliver synergistic protection against acute lung injury evaluated. The outcomes designated that the treatment by combination showed the same activity as dexamethasone (DEX), both of which downregulated the expressions of IL-6, TNF-α, MDA as well as MPO, lung structural injuries reduced, and GSH upregulated. Also, NF-κB p65 activation was inhibited by combination treatment and DEX. The study revealed that treatment by combination with lycopene and matrine showed protective activity on LPS-caused ALI mouse model [147].

The study aims to check the hypothesis that Sarcandra glabra in the combination of lycopene protects rats from LPS-caused acute lung injury (ALI). The study revealed that *Sarcandra glabra*, in combination with lycopene, protects rats from LPS-induced ALI [41]. 

The role of supplementation of lycopene on lung histopathology, and inflammation, and systemic DNA damage in lung injury model was investigated. Based on results, it was concluded that supplementation lycopene decreases inflammatory and lung injuries, regardless of the linked ventilatory mode. Also, lycopene improved oxygenation as well as decreased DNA damage when protecting conventional mechanical ventilation was used [148]. Lycopene was assessed to treat pulmonary fibrosis induced by bleomycin (BLM) in rats. The results showed that the lung coefficients in the lycopene + BLM-treated group were reduced, as well as the extents of alveolitis and PF. The concentrations of TNF-α, NO, and malondialdehyde in plasma, as well as the expression of TNF-α in lungs, were reduced, while the plasma superoxide dismutase activities increased in the lycopene + BLM-treated group as compared with the BLM-treated group [149].

### 4.12. Effects of Lycopene in Reproductive System-Related Pathogenesis

The role of lycopene in reproductive system-related pathogenesis is mediated by multiple mechanisms (Figure 5). It employs strong antioxidant effects and anti-inflammatory properties through the reduction in oxidative stress and regulates inflammatory pathways involved in reproductive ailments. These combined activities contribute to their protective role in prostate disorders and other reproductive health issues. The role of lycopene in reproductive system-associated pathogenesis is mediated through different mechanisms (Table 6). The study results showed noteworthy increases in DNA damage, ROS levels, MDA, and alterations in sperm parameters, including motility and reduced concentration in the varicocele group. Lycopene treatment improved sperm concentration-enhanced total antioxidant capacity and CAT activity and reduced ROS levels, DNA damage, and MDA as compared to the varicocele group. Overall, these results indicate that lycopene, particularly at a dose of 10 mg/kg, protects against oxidative stress-induced sperm damage by reducing ROS levels and enhancing antioxidant defenses [150]. The protective role of lycopene against IR-induced testicular damage in mice was examined. It was noticed that lycopene remarkably increased sperm motility and reduced sperm abnormalities in mice following exposure of IR [151]. Peranandam Tamilselvan et al. 2024 demon stated that lycopene (10 mg/kg b.w) administration orally with Bisphenol A (BPA)-intoxicated rats diminished the testicular toxic condition; morphological and biochemical changes were taken back to normal [152]. Moreover, lycopene ameliorates the changes that are caused by BPA [152].

### 4.13. Role in Skin Health

A study was conducted to assess the effects of a lycopene-rich tomato nutrient complex (TNC) against ultraviolet (UVA/B and UVA1) radiation at the molecular level. The results confirmed that TNC effectively inhibited the UVA1 and UVA/B-induced upregulation of heme oxygenase-1, intercellular adhesion molecule-1, and matrix metallopeptidase-1 mRNA expression [153]. A study investigated whether tomato paste, rich in lycopene, could protect human skin against ultraviolet radiation (UVR)-induced damage. The outcomes showed that the mean ± SD erythemal dose (ED_30_) was suggestively higher in the tomato paste group compared to the control group. When it comes to presupplementation, UVR induced an increase in MMP-1 and a reduction in fibrillin-1. Moreover, postsupplementation, UVR-induced MMP-1 was reduced in the tomato paste, whereas the UVR-induced decrease in fibrillin-1 was similarly abrogated in both groups, and an increase in pCI deposition was noted following tomato paste [154].

### 4.14. Effect of Lycopene on Bone Health

The lycopene effects on bone health, mediated through various mechanisms, are summarized in Table 6. An experiment was conducted to evaluate the protective effect of lycopene against glucocorticoid-induced osteoporosis in a rat model. A study found that osteoporosis caused by glucocorticoids can be partly lessened by co-administration of lycopene [155]. A study established a diabetic rat model and administered lycopene to see its effect on diabetic osteoporosis. The results exhibited that lycopene treatment restored bone mechanical and micro-CT parameters and enhanced bone mineral density of diabetic rats. After lycopene, serum bone turnover marker levels were downregulated [156]. The role of lycopene on the differentiation and function of human osteoclasts and osteoblasts was evaluated. It was noticed that lycopene seems to promote an anabolic state of bone metabolism, stimulation of osteoblastogenesis, and inhibition of osteoclastogenesis, which may contribute to the promotion of proper bone tissue health status [157]. The study was planned to evaluate the role of lycopene on bone cells and in the microarchitecture of ovariectomized rats. Results exhibited that lycopene promoted an increase in ALP in situ detection as well as a noteworthy increase in mineralized nodule deposition as compared with the OVX group. The lycopene administration increased the total number of osteocytes and osteoblasts. Furthermore, it reduced the number and volume of osteoclasts and decreased the volume of osteocytes [158].

**Table 6:** The role of lycopene in various pathogenic conditions, including the experimental models, the doses administered, and the key findings.

Activity	Study Model	Dose	Findings	Refs.
Anti-colitis	DSS-induced colitis in mice	12 mg/kg	° Lycopene attenuated colitis.	[137]
	Acetic acid-induced ulcerative colitis in rats	5 mg/kg	° Lycopene ameliorative effect against destructive changes caused by UC.	[138]
	Acetic acid-induced ulcerative colitis	10 mg/kg	° Lycopene reduces colitis in rats by decreasing inflammation and apoptosis.	[139]
Renoprotective	High fat diet caused kidney injury in rats	25 and 50 mg/kg	° Lycopene treatment reverses HFD-mediated changes.	[144]
	Kidney damage in rats	5 mg/kg	° Lycopene kidney damage.	[145]
Role in asthma	Ovalbumin-induced murine asthma model	8 or 16 mg/kg	° Infiltration of inflammatory immunocytes inhibited by lycopene.	[146]
Role in pulmonary fibrosis in rats	Bleomycin-induced experimental pulmonary fibrosis in rats	5 mg/kg	° Lycopene alleviates pulmonary fibrosis.	[149]
Role in reproductive system	Ionizing radiation-induced testicular damage in mice	20 mg/kg	° Lycopene alleviates testicular damage.	[151]
Role in bone health	Glucocorticoid-induced osteoporosis in rat	30 mg/kg	° Osteoporosis was to some extent minimized by lycopene co-administration.	[155]

### 4.15. Wound Healing Property

Natural products and bioactive compounds have demonstrated the ability to enhance wound healing by reducing inflammation and promoting the regeneration of tissues. A study was made to find the wound healing properties of lycopene emulgel (LE). Wound healing action was measured in diabetic rats. Treatment of rats with LE topical application showed a significant decrease in wound closure [159]. The lycopene was intercalated into the dipalmitoyl-phosphatidylcholine (DPPC) liposomes loading with tobramycin (TOB) and additionally formulated with HAMA hydrogel (LT NPs@Gel) to make an innovative multifunctional antibiotic wound dressing. In an infected diabetic wound model on rat, the local administration of LT NPs@Gel meaningfully helped wound healing within 14 days [160].

### 4.16. Role of Lycopene in Oral Health

Medicinal plants and their bioactive compounds hold anti-microbial as well as anti-inflammatory activities, which contribute to the maintenance of oral health. A study was made to evaluate the impact of systemically administered lycopene as an adjunct to scaling and root planing in patients with gingivitis and periodontitis. The results reveal that lycopene is an effective adjunctive treatment to full-mouth scaling and root planing (SRP) in patients with periodontal disease [161].

The study was designed to compare the effectiveness of lycopene gel and minocycline hydrochloride microspheres (ARISTIN) when used as adjuncts to nonsurgical therapy for the treatment of periodontitis. Both ARISTIN and lycopene treatments exhibited better gains in attachment than the control treatment, meaningfully. Lycopene gel, as well as ARISTIN, offers nearly equal improvement in both biochemical and clinical parameters of periodontitis [162]. A study was conducted to assess the efficiency of oral lycopene therapy in patients with oral submucous fibrosis. Based on findings, it was reported that lycopene may serve as a first-line therapeutic choice in the oral submucous fibrosis early management [163]. The study evaluated the efficiency of lycopene in treating oral leukoplakia and compared two dosage levels with a placebo. A total number of 58 patients, clinically and histologically diagnosed with oral leukoplakia, were assigned into the following three groups: Group A received 8 mg lycopene/day, Group B received 4 mg daily, and Group C received a placebo. The results showed that lycopene may be a safe and effective choice for management of oral leukoplakia [164].

### 4.17. Anti-Arthritis Effects

The worldwide prevalence of arthritis is significant, including over 100 different types [165,166]. Medicinal plants, including lycopene, play a role in combating arthritis through various mechanisms. The effect of lycopene on the inflammation of chondrocytes, as well as the mouse osteoarthritis model, was investigated. It was reported that lycopene improves the degradation of the extracellular matrix and inhibits inflammatory factor expression. The animal studies proved that lycopene lowered the Osteoarthritis Research Society International scores in the knee, suggesting its potential to hinder the onset and progression of osteoarthritis in mouse [167].

### 4.18. Anti-Microbial Effect

Flavonoids, phenolic acid and other natural compounds hold remarkable antimicrobial properties, functioning through a variety of effective mechanisms. These include disrupting membranes, inhibiting biofilm formation, hindering enzymes involved in metabolic pathways, destroying cell walls, and interfering with the replication of genetic material. The study was made to evaluate the antiviral potential of lycopene against HSV-1 infection compared with acyclovir. Noteworthy antiviral activity of lycopene was noticed against HSV-1 at a concentration of 25 µg/mL. This study demonstrated that lycopene revealed substantial antiviral activity as well as anticytotoxic activity against HSV-1 infection in vitro [168]. The capability of lycopene to apoptosis induction and the mechanism by which it controls apoptosis were evaluated. It was noticed that lycopene caused antifungal effects during the early as well as late stages of apoptosis in Candida albicans. During apoptosis, increased ROS and treatment by lycopene caused mitochondrial dysfunction and intracellular Ca(2+) overload. Overall, lycopene caused its antifungal role against C. albicans through induction of apoptosis by mitochondrial dysfunction and ROS production [169]. It is reported that lycopene exerts a powerful antifungal action on the serum-induced mycelia of C. albicans. The results indicated that lycopene caused noteworthy membrane damage and inhibited the normal budding process, resulting from the destruction of membrane integrity [170]. A study assessed the antimicrobial combination effect of lycopene, allicin, and quercetin extracted from tomatoes, garlic, and onions, respectively, against MRSE, MRSA, and multi-drug-resistant *Escherichia coli*. The combination of allicin, lycopene, and quercetin at a ratio of 2:3:2 µg exhibited the highest activity at both 24 h and 48 h marks, resulting in mean inhibition zones from 20 ± 1.26 mm to 44 ± 2.77 mm. Additionally, a concentration of 5:1 µg for lycopene and quercetin produced mean inhibition zones from 16 ± 1.01 mm to 25 ± 1.58 mm across all tested bacteria. For the typed culture isolates, the mean inhibition zones were recorded as 8 ± 0.50 mm to 20 ± 1.27 mm when tested singly, and 8 ± 0.50 mm to 22 ± 1.39 mm when tested in combination [171].

The evidence from above studies presented indicates that lycopene plays a significant role in various pathological conditions by modulating multiple biological activities, including the downregulation and upregulation of key molecular pathways, reduction in oxidative stress, suppression of inflammation, inhibition of microbial growth and biofilm formation. Ultimately, these mechanisms contribute to the inhibition of disease development and progression.

## 5. Synergistic Effects of Lycopene When It Is Used with Other Drugs or Bioactive Compounds

Lycopene has been revealed to show synergistic activities when used in combination with other drugs/compounds. Recent studies have demonstrated that combination strategies of lycopene with natural phytochemicals, trace elements, and chemotherapeutic agents enhance therapeutics’ efficacy through synergistic pharmacological interactions. To provide clearer organization, the combinational approaches are characterized below based on the type of co-administered agent.

i. Combination with phytochemicals and vitamins

**Figure 6:**
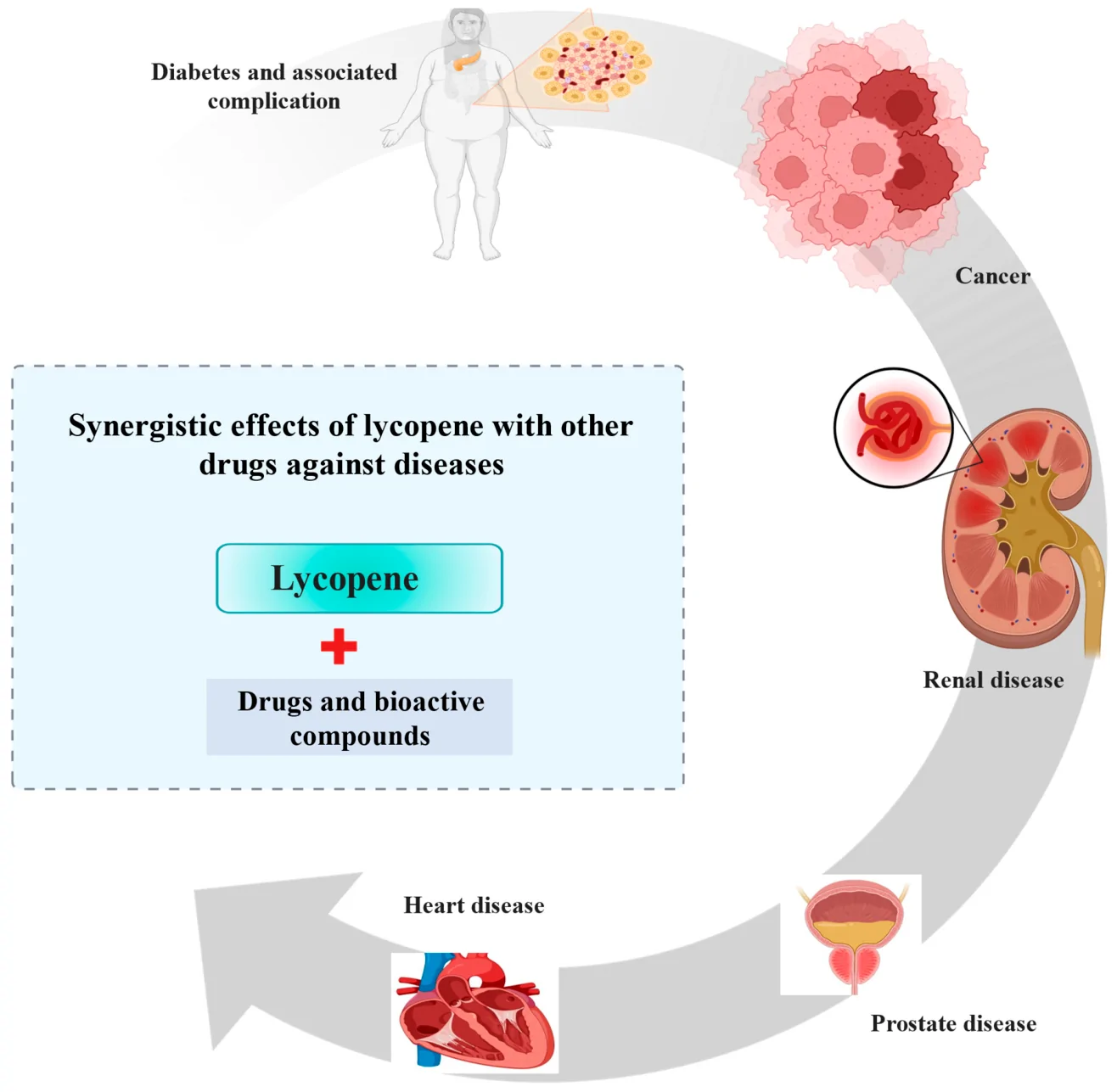
The synergistic properties of lycopene in combination with other compounds in diseases. Lycopene, when combined with drugs and other bioactive compounds, and increased their efficacy and finally manages pathological conditions. The figure was created in https://BioRender.com.

The combination of lycopene with phytochemicals and vitamins has revealed promising synergistic activity. As demonstrated in Figure 6, such synergism improves its functional efficacy in various physiological circumstances. The detailed combinations and their effects are further described in Table 7. In this discussion, we explore the synergistic effects of lycopene with phytochemicals and vitamins in detail.

The study aimed to measure the inhibitory effect of the combination of lycopene as well as curcumin on benign prostatic hyperplasia. The combination treatment meaningfully reduced prostate hyperplasia and lessened benign prostatic hyperplasia pathological characteristics in rats [172]. The role of lycopene, curcumin, and irradiation upon oral squamous cell carcinoma was investigated. A study found that lycopene and curcumin enhance cytotoxic activity in the PE/CA-PJ15 cell line and decrease cell migration capacity, whereas the combination of lycopene or curcumin with irradiation causes synergic activity [173]. A study based on diabetic rats reported that treatments with mixtures of lycopene as well as curcumin or bixin showed combined activity, declining biomarkers of carbohydrate as well as lipid disturbances (curcumin activity); the HDL levels increased (carotenoids effects) and mitigated oxidative stress (carotenoids and curcumin activity). The combined activity also caused LDL oxidation prevention, in that way mitigating the cardiovascular risk in diabetes [174]. The effects of lycopene combined with quercetin and curcumin on chronic prostatitis/chronic pelvic pain syndrome were examined. The study found that the combination of lycopene, curcumin, and quercetin was more effective than any of the three agents used individually in treating chronic prostatitis/chronic pelvic pain syndrome [175]. The cardiac and neuroprotective activity of the combination of lycopene and quercetin was examined in d-galactose-induced oxidative stress in mice. Results exhibited that the lycopene–quercetin combination ameliorates histopathological damage in the heart and hippocampus. The heart and hippocampal mRNA level of Nrf2 expression was upregulated, and the antioxidant genes related to Nrf2, including HO-1 and NQO1, were elevated [176]. The ability of the two compounds (lycopene and vitamin E) to reduce tumor growth of human prostate cancer was examined. It was reported that combined treatment with lycopene as well as vitamin E suppressed the growth of prostate tumors and increased median survival time [177].

ii. Combination with Chemotherapeutic Agents

Combinations with chemotherapeutic drugs are investigated. These synergistic interactions improve efficacy outcomes. A study on cervical cancer described that lycopene acts synergistically with cisplatin to inhibit the growth of cervical cancer cells. The findings proposed that lycopene enhances the sensitivity of cervical cancer cells to cisplatin by downregulating Bcl-2 expression, upregulating Bax expression and reducing cell viability [178]. Another study on colon cancer proved that a combination of lycopene and 5-fluorouracil (5-FU) enhanced 5-FU-induced apoptosis and promoted necrosis. In the wound healing assay, the combination treatment also inhibited cell migration more effectively than treatment with 5-FU alone [179]. A breast cancer-based study described that lycopene synergistically increased quinacrine action and inhibited Wnt-TCF signaling in cancer cells [127]. The study was made to deliver data support for lycopene and enzalutamide combination in the treatment of castration-resistant prostate cancer. The outcomes advocate that the enhanced antitumor effects of enzalutamide through lycopene might be associated with the decrease of AR protein levels by lycopene-mediated inhibition of AKT/EZH2 pathway, which may offer a novel approach to improve the efficiency of enzalutamide in castration-resistant prostate cancer [180].

A study based on Solid Ehrlich Carcinoma was made to evaluate the antitumor potential of combination of sorafenib as well as lycopene. The results confirmed that the combination was higher than sorafenib and lycopene only in the suppression of cancer cell viability, early cell cycle arrest, and increasing apoptosis and cell autophagy. Moreover, combination treatment reduced inflammation and enhanced apoptosis, as observed by the tumor’s weight and decreased volume. The anti-lung-metastatic role of lycopene in combination with sorafenib was determined. The study based on findings concluded that lycopene with sorafenib combination additively inhibits the lung metastasis of tumor, demonstrating lycopene has a role as an adjuvant for sorafenib in the treatment of cancer [181].

iii. Other Combination Strategies

Additional combinations have been explored to further improve therapeutic performance. These approaches aim to achieve higher efficacies through modulation of biological activities. The ameliorative effect of lycopene and/or N-acetylcysteine in rats with hepatic and renal toxicity caused by cisplatin (CP) was determined. The activities of ALT, AST, and APK and levels of urea, creatinine, and lipids increased after injection of CP in the serum. Furthermore, CP decreased levels of protein, HDL cholesterol, and albumin and increased malondialdehyde, resulting in a decrease in antioxidant enzymes. Administration of lycopene and N-acetylcysteine alone or in combinations ameliorated hepatorenal toxicity as well as apoptosis induced by CP [182]. Another study finding demonstrated that a combination of lycopene as well as selenium was more powerful than supplementation of either agent only in prevention of testicular damage induced by cisplatin [183].

Overall, the studies demonstrate that lycopene exhibits strong synergistic effects when combined with other bioactive compounds/drugs, resultant in improved anticancer, anti-inflammatory, and antioxidant activities. These combinations improve therapeutic outcomes by modulating various biological activities. Collectively, this evidence highlights the potential of lycopene-based combination strategies as promising approaches for improving therapeutic efficacy.

**Table 7:** The table described the therapeutic activities, together with anticancer, anti-inflammatory, and protective effects on testes and prostate.

Drugs/Compounds	Activity	Finding	Refs.
Curcumin	Effect on benign prostatic hyperplasia	° The combination treatment attenuated prostate hyperplasia and alleviated BPH pathological features.	[172]
Curcumin	Effect on oral squamous cell carcinoma	° Curcumin as well as lycopene reduce cell migration capacity.	[173]
Curcumin	Effect on diabetic complication	° Curcumin and carotenoid mixtures act as therapy for diabetic complications.	[174]
Quercetin and curcumin	Effect on chronic prostatitis/chronic pelvic pain syndrome	° Lycopene combined with quercetin and curcumin alleviates inflammatory response and oxidative stress.	[175]
Vitamin E	Effect on prostate cancer	° Combination treatment inhibits the growth of cancer significantly.	[177]
Cisplatin	Effect on cervix cancer	° The combination prevents the growth of HeLa cells.	[178]
5-Florouracil	Effect on colon cancer	° Lycopene and 5-FU treatment inhibited cell migration.	[179]
Quinacrine	Effect on breast cancer	° The lycopene synergistically increases QC anticancer activity.	[127]
Sorafeni	Effect on Solid Ehrlich Carcinoma	° The combination reduced inflammation, tumor’s volume and weight.	
Sorafenib	Effect on lung carcinoma	° Lycopene and sorafenib significantly inhibit tumor metastasis.	[181]
N-acetylcysteine	Effect on hepatorenal toxicity	° Administration of combinations ameliorated hepatorenal toxicity.	[182]
Cisplatin	Effect on testicular toxicity	° Selenium and lycopene supplementation reduced testicular toxicity.	[183]

## 6. Lycopene Bioavailability and Strategies to Improve Efficacy of Lycopene in Pathogenesis

Lycopene is a significant compound whose role in various pathological conditions has been established by different mechanisms including antioxidant, anti-inflammatory, anti-apoptotic, and cytoprotective activities. Lycopene causes beneficial properties chiefly by scavenging free radicals, modulating inflammatory mediators, reducing oxidative stress, and maintaining tissue integrity. Despite its use, it is restricted by several challenges such as low solubility, low permeability, rapid release, and low bioavailability (demonstrating that only a small portion of the administered substance actually reaches the target organs). Lycopene holds a molecular structure comprising 13 double bonds, of which 11 are conjugated, contributing to its potent antioxidant properties as well as characteristic deep red coloration [184,185]. However, these conjugated double bonds are highly susceptible to oxidative and environmental stressors such as light, heat, and acid, which can induce degradation or rearrange the lycopene structure to different spatial *cis* configurations from *all-trans*-isomers [186]. Such structural changes may reduce the biological activity of lycopene [186,187]. The study was performed to measure the long-term human lycopene bioavailability in plasma as well as skin after a single dose of ^14^C-lycopene and to profile the metabolites formed. Based on findings, the study concluded that lycopene was broadly isomerized after dosing as well as quickly metabolized into polar metabolites excreted into urine. In skin, lycopene or its metabolites were noticed for up to 42 d [188].

To overcome these limitations, drug delivery systems based on nanotechnology have been developed to improve stability, as well as therapeutic efficiency of lycopene. Various nanoformulations, including nanoparticles, nano-emulsions, liposomes, micelles, and lipid nanoparticles, have shown auspicious results in improving lycopene solubility and bioavailability. Study results reported that the nanostructured lipid carrier (NLC) showed better permeation and caused significant cytotoxicity due to superior penetration and more bioavailability [189]. Lycopene nnanoformulationand its role in pathogenesis is outlined in Table 8. The results of antioxidant activity using DPPH and ABTS assays revealed that nano-formulation of lycopene increases the scavenging activity as compared to lycopene. Also, both lycopene and lycopene-NPs exhibited antifungal activity against the tested fungal species and antibacterial activity against all tested bacteria. The MTT assay showed cytotoxic action against three cancerous cell lines [190]. The study stated that the lycopene nano-suspension had a mean particle size of 100 ± 4.50 nm and a polydispersity index of 0.04, indicating a uniform and stable nananoformulationIn vivo antidiabetic studies confirmed that oral administration of lycopene nanoparticles led to a substantial reduction in elevated blood glucose levels and improved biochemical parameters in a dose-dependent manner, along with noteworthy antioxidant activity [191]. The study was designed to evaluate the antioxidant, anti-inflammatory, and antimicrobial properties of lycopene (Lyc), lycopene selenium nanoparticles (Lyc-Se-NPs) as well as selenium nanoparticles (Se-NPs). The CLSM confirmed that *S. aureus* treated by sub-MICs of Lyc-Se-NPs exhibited a substantial biofilm formation reduction. Additionally, the group treated by 50 mg of this formulation exhibited the fastest rate of wound healing [192]. Another study on polymeric nanoparticle-based lycopene formulation on prostate cancer cells and its result noticed that good anti-prostate cancer activity is based on in vitro cytotoxicity [193]. The effects of lycopene in the form of nanoliposomes (L-LYC) on ischemic brain injury were explored. It was reported that L-LYC increased the lycopene content in serum and the brain. Moreover, L-LYC reduced cerebral infarction and improved the neurobehavior of the rats more effectively than LYC. The results confirmed that nano-liposomal encapsulation meaningfully improved LYC efficiency in providing neuronal protection against I/R injury [194]. A study was made to evaluate the effects of lycopene, administered in two different pharmaceutical forms, against kidney damage caused by methotrexate in rats. The study evaluated serum biochemical markers (creatinine and urea levels), tissue oxidative damage markers, as well as histopathological alterations in the kidneys after the systemic administration of both lycopene dissolved in corn oil and lycopene encapsulated in nanoliposomes. The outcomes showed that the application of both forms of lycopene, given together with methotrexate, improved serum urea, creatinine levels, reduced oxidative damage markers, and suggestively reversed structural changes in kidney tissue [195]. The phosphatidylserine-chitosan nanoparticles of lycopene alleviated experimental streptozotocin-induced oxidative stress, thereby improving behavioral and cognitive anomalies, while enhancing the antioxidant-related activity of the enzyme [196]. The lycopene was loaded into lipid nanoparticles to improve penetration and pharmacological properties. The solid lipid nanoparticles (SLNs) comprising lycopene were made and anti-tyrosinase activities were studied. In defining the anti-tyrosinase effects of LYC-SLNs, a significant decrease in cellular tyrosinase activity as well as melanin and ROS levels was noticed. It was observed that LYC-SLNs reduced melanin production with nominal toxicity against melanoma cells [197]. The in vitro cytotoxicity study of lycopene loaded whey protein isolate nanoparticles (LYC-WPI-NPs) was assessed against -7 breast cancer cells MCF. The WPI-NPs improve the oral lycopene bioavailability through controlling its release from nano-formulation as well as facilitating its absorption. Prophylactic anticancer efficiency of LYC-WPI-NPs was assessed thereafter on breast cancer animal model. Conclusively, it might be relatively reasonable that lycopene loaded protein nanoparticles are capable to improve the biopharmaceutical attributes of lycopene as well as confirmed prophylactic anticancer activity, tumor proliferation decreased and the survival rate of treated animals increased [198].

The study was made to examine the effect of some precarious variables such as chitosan concentration, sodium tripolyphosphate concentration (STPP) as well as stirring time on physicochemical characteristics of lycopene-loaded chitosan nanoparticles. Based on findings, the study concluded that lycopene-loaded chitosan nanoparticles may demonstrate good potential for drug delivery system development by cellular accumulation enhancement of lycopene with chitosan [199].

Several nanoformulations have been developed to enhance its solubility, stability, bioaccessibility, and anticancer efficacy but these nanosystems differ considerably in their pharmacological potential and translational potential. The polymeric nanoparticles appear promising because they demonstrated improved solubility and increased bioavailability. Overall, lipid-based nanosystems, particularly liposomes, and solid lipid nanoparticles represent the most promising strategies for enhancing lycopene solubility, bioavailability, and efficacy.

Overall, it is reported that lycopene exhibits low bioavailability due to its poor solubility, limited absorption, and rapid degradation. These limitations meaningfully affect its therapeutic efficacy. To overcome these challenges, several advanced delivery strategies have been developed to improve lycopene solubility, bioavailability, and targeted cellular uptake, thereby enhancing its pharmacological and therapeutic potential.

**Table 8:** Nanoformulations based on lycopene reported various activities as presented in the table below.

Nanoformulation	Activity	Findings	Refs.
Lipid-based nanoformulation of lycopene	Anti-breast cancer	° Formulation showed good permeation and caused more cytotoxicity.	[189]
Crystalline nano-suspension of lycopene	Antidiabetic activity	° The nano-preparation exhibited powerful for antidiabetic effects.	[191]
Lycopene selenium nanoparticles	Antibacterial, antioxidant, and anti-inflammatory	° Promising antibacterial, antioxidant, and anti-inflammatory properties noticed by this formulation.	[192]
Lycopene-loaded polymeric nanoparticles	Anti-prostate cancer	° This formulation shows better anti-prostate cancer potential.	[193]
Nanoliposomes of lycopene	Neuronal protection	° Formulation improved lycopene effectiveness in providing neuronal protection.	[194]
Nanoliposome-encapsulated lycopene	Reduces kidney dysfunction	° The nanoliposome-encapsulated lycopene more capably decreases kidney dysfunction.	[195]
Lycopene-loaded solid lipid nanoparticles	Anti-melanogenesis	° Formulation reduced melanin production.	[197]
Lycopene-loaded whey protein isolate nanoparticles	Anticancer activity	° Formulation caused significant decrease in tumor proliferation.	[198]

## 7. Safety and Toxicity of Lycopene

The assessment of the safety level of natural compounds and their bioactive components is vital for their use as health-promoting agents. Understanding the safety profile together with adverse effects and long-term consequences is vital before using these substances. A safety assessment based on in vitro studies, animal models, and clinical trials is indispensable, and such studies help identify dose-dependent toxicity. So, wide-ranging toxicological assessment and safe, effective dosing are important to confirm that natural compounds are safely used in therapeutic health approaches.

Lycopene obtained from natural dietary sources such as tomatoes as well as other red fruits is generally considered safe and well-tolerated in humans. However, the safety profile of concentrated lycopene supplements may differ from that of dietary intake because supplementation can result in substantially higher doses and bioavailability. A study indicated that doses of up to 3000 mg/kg of formulated lycopene demonstrated no effects on body weight, body weight gain, food consumption, hematology, clinical chemistry, urinalysis, or ophthalmoscopic parameters in rats. The no-observed-adverse-effect level (NOAEL) for the study was noted to be 3000 mg/kg body weight per day for both lycopene CWD and Lyco Vit. These results show that neither product produced any substantial toxicological effects, even at very high dose levels [200]. The results of the study do not provide any sign of toxicity of lycopene at dietary levels up to 1.0%. The no-observed-adverse-effect level at the highest dietary concentration was tested (1.0% in the diet) [201]. Doses ranging from an optimal 12 mg/day up to very high levels of 150 mg/100 g have shown no toxic effects in the populations studied in the literature [202].

Based on no-observed-effect level (NOAEL) data from toxicity studies of synthetic lycopene formulations, the European Food Safety Authority (EFSA) recognized an acceptable daily intake (ADI) of 0.5 mg/kg body weight per day [203]. In the NHANES 2007–2016, a U-shaped association was recommended, with the intake not higher than 10 mg per day designated as the most beneficial [204]. However, reported lycopene intake varies across populations, with lower consumption in Spanish adults (1.64 mg/day) compared with higher intakes observed in France and the United Kingdom (4.43–5.01 mg/day) [20,205]. In the United States, average daily intake exceeds 7 mg/day [21]. Epidemiological studies suggest that a daily lycopene intake ranging from 2 to 20 mg may be beneficial in the prevention and management of various diseases [206].

A case report showed a woman diagnosed by lycopenemia due to prolonged excessive intake of lycopene as daily consumption of approximately 2 L of tomato juice over several years. The patient had lycopene accumulation in the liver although there were no signs of liver dysfunction and her skin displayed a deep orange discoloration. Remarkably, the lycopenodermia was resolved within three weeks after she ceased her intake of tomato juice [207]. The study was designed to inspect the relations between the intake of dietary carotenoids and associated compounds by pregnant women and the risk of them developing preeclampsia (PE). These outcomes indicate that a high intake of β-cryptoxanthin, total carotenoids, β-carotene, lycopene, and lut-zea may be related to a low risk of developing PE [208]. Although reported adverse effects are uncommon, excessive or long-term supplementation may raise safety concerns, mainly in pregnant women and children.

## 8. Lycopene-Based Clinical Trials

Clinical trials examining the effectiveness and safety of lycopene remain relatively limited. Although some studies suggest that lycopene may offer potential health benefits, robust clinical evidence is still needed to explore pharmacokinetics, optimum dosages, and benefit of lycopene in disease management.

Some of the clinical study trials based on lycopene are discussed to show its role in disease management. Clinical studies evaluating the therapeutic effects of lycopene supplementation or lycopene-enriched formulations in different patient populations are compiled in Table 9. Clinical studies evaluated the therapeutic effects of lycopene supplementation or lycopene-enriched formulations in different patient populations. A total number of 33 postmenopausal women aged 50–60 years were given 7-day dietary records and blood samples. Serum samples were obtained to quantify serum lycopene, lipid peroxidation, protein thiols, cross-linked N-telopeptides of type I collagen, NTx and bone alkaline phosphatase. The outcomes exhibited that groups with higher intake of lycopene, as determined from the dietary records, had higher serum lycopene. A higher serum lycopene was noticed to be related to a low NTx and groups of higher serum lycopene had lower protein oxidation. These outcomes advise that the lycopene decreases oxidative stress as well as the levels of bone turnover markers in postmenopausal women and might be valuable in dropping the osteoporosis risk [209]. A study was conducted to investigate whether polymorphisms in the paraoxonase 1 (PON1) genotypes affected the relationship among lycopene, bone turnover markers, and parameters of oxidative stress. Blood samples from 107 women were examined for serum carotenoid concentrations, N-telopeptide of type I collagen, bone-specific alkaline phosphatase and oxidative stress parameters. The findings indicate that PON1 polymorphisms modify the relationship between serum levels of lycopene and both oxidative stress parameters and bone turnover markers, suggesting they affect the risk of developing osteoporosis [205,210]. A study carried out a pilot controlled clinical study to confirm the possibility of an approach for bone loss prevention by the intake of a lycopene-rich tomato sauce in 39 postmenopausal women. It was noticed that substantial bone density loss was not noticed in women taking the tomato sauce, whereas the control group had bone loss. Tomato sauce intake caused a better bone alkaline phosphatase reduction than the control [211].

The study was made to assess the clinical performance of lycopene-enriched virgin olive oil used to treat the condition. A total of sixty patients by BMS were arbitrarily separated into the following two groups: Group I treated with lycopene-enriched virgin olive oil and Group II treated with a placebo. The topical lycopene-enriched virgin olive oil is a safe and effectively similar way in which the placebo treats BMS patients [212]. Another study was made to assess the effect of lycopene-enriched virgin olive oil in spray form used to treat xerostomia patients. Based on findings, the study concluded that the lycopene-enriched virgin olive oil topical application and its placebo counterpart improved xerostomia-associated symptoms [213]. The role of supplementation of lycopene on spermatogram and seminal oxidative stress was checked. A total 44 infertile men with oligozoospermia were arbitrarily separated into two groups as follows: the experimental group was lycopene-supplemented (25 mg), and the control group got placebo for 12 weeks. It was concluded that the supplement of lycopene improves sperm parameters as well as oxidative stress biomarkers in oligozoospermia infertile men [214]. The role of lycopene (LycoRed) supplementation in promoting bone and cardiovascular health was assessed in healthy postmenopausal women. In this study, 100 participants were assigned to receive either LycoRed 8 mg (57 women) or placebo (43 women) for six months. The outcomes exhibited that LycoRed supplementation increased lycopene levels and P1NP, along with a nonsignificant decrease in β-CTx levels. These results recommend that lycopene supplementation may help protect against osteoporosis in Indian postmenopausal women, as shown by directional changes in surrogate biochemical markers [215]. Another study examined the role of lycopene-restricted diet on oxidative stress biomarkers as well as bone turnover markers in postmenopausal women. The findings proposed that regular dietary intake of lycopene may play a vital antioxidant role in reducing bone resorption and maintaining bone health. Consequently, adequate lycopene consumption contributes to lowering the risk of osteoporosis in postmenopausal women [216]. Clinical studies advocate that lycopene supplementation may deliver health benefits, although evidence remains limited. However, while some studies report positive outcomes, others found lycopene-enriched formulations to be no more effective, highlighting the need for larger, well-designed clinical trials to establish optimum dosages, safety, mechanism and therapeutic value in disease management.

## 9. Conclusions, Limitations and Future Directions

Lycopene, a naturally occurring red-orange carotenoid mainly found in tomatoes, has gained significant attention for its potent antioxidant, anti-inflammatory, and cytoprotective properties. Accumulating evidence designates that lycopene exerts a beneficial role in a wide range of pathogenesis, together with liver and lung diseases, cardiovascular and neurodegenerative disorders, reproductive and digestive dysfunction, skin disorders, and cancer. Among the proposed mechanisms in the management of pathogenesis, the most promising evidence supports lycopene’s antioxidant and anti-inflammatory activities, particularly via reduction in oxidative stress, ROS, inflammatory mediators, and other biological processes. Preclinical studies based on cancer demonstrated that lycopene has valuable chemopreventive and therapeutic value through regulation of cell proliferation, induction of apoptosis, autophagy, inhibition of angiogenesis, cell cycle arrest, and PI3/Akt pathways. In addition, synergistic combinations of lycopene with other bioactive compounds and cancer drugs have shown enhanced pharmacological efficacy compared with lycopene alone, particularly by improving protection against oxidative stress, inflammation, cellular injury, and cancer.

Recent advances in nanoformulation strategies, including liposomes, solid lipid nanoparticles, and polymeric nanoparticles, also appear promising for overcoming the major limitations associated with lycopene, especially its poor water solubility, low bioavailability, chemical instability, and rapid degradation. Among these systems, lipid-based nanosystems demonstrate the greatest translational potential because of their ability to improve solubility and targeted delivery.

However, despite supportive experimental and preclinical studies, the current evidence base for clinical/translational applications remains inadequate. Most existing studies are limited to in vitro as well as animal models, whereas large-scale, controlled clinical trials are still scarce. Substantial gaps remain about the optimal dosage, long-term safety, pharmacokinetics, bioavailability, drug interactions, and therapeutic efficacy of lycopene in different pathologies. Moreover, several novel nanoformulations and combination approaches have shown only preliminary laboratory-scale effects and require further validation before clinical translation. Future detailed research, based on well-designed clinical studies, comparative evaluation of nanoformulations, and comprehensive mechanistic investigations, is needed better to understand the clinical value of lycopene in disease management.

## Figures and Tables

**Table 1:** Hepatoprotective actions of lycopene. It outlines study models, the administered doses of lycopene, and key findings which include reduction in liver enzymes, histopathological changes associated with hepatic damage and modulation of other activities.

Study Model	Doses	Findings	Ref.
D-Gal-/LPS-induced hepatitis rat model	10 mg/kg	° Lycopene stabilizes lipoprotein levels and lipid metabolism	[47]
Atrazine-induced hepatotoxicity mice model	5 mg/kg	° Lycopene protects liver toxicity by cytochrome P450 enzyme modifications	[48]
Tramadol-induced liver toxicity rat model	10 mg/kg	° Lycopene reduced the hepatotoxic effects	[49]
D-GalN-/LPS-induced hepatitis rat model	10 mg/kg	° Lycopene combated oxidative damage and protected antioxidant defense	[50]
Hepatic ischemia/reperfusion injury rat model	2.5 and 5 mg/kg	° ALT, AST, LDH and MDA values decreased by lycopene	[51]
CCL4-induced liver injury rat model	0.35, 0.65 and 1.30 mg/kg	° The level of markers for hepatic integrity was close to the controls by lycopene	[52]
CCL4-induced hepatic fibrosis rat model	10 mg/kg	° Lycopene reduces hepatic fibrosis	[53]
Paracetamol-induced liver damage rat model	4 mg/kg	° Lycopene treatment reduced serum transaminase levels	[54]
Acetaminophen-induced hepatotoxicity mice model	10 and 100 mg/kg	° Lycopene pretreatment improves hepatotoxicity	[55]

**Table 2:** Anti-diabetic effects of lycopene. This table summarizes the evidence on the anti-diabetic effects of lycopene, including the study models used, the administered doses, and the major findings.

Study Model	Dose	Findings	Refs.
STZ-induced DM in rats	10 mg/kg	° Lycopene decreased the pathological changes and increased serum insulin levels.	[60]
STZ-induced diabetics in rats	10, 20 and 40 mg/kg	° Lycopene reduced the blood glucose concentration.	[61]
Streptozotocin-induced diabetic in rats	10 mg/kg or 20 mg/kg	° Lycopene has caused an effect on anti-diabetes and regulates the metabolism of glycolipids in diabetic rats.	[62]
STZ-induced diabetics in rats	2.5 mg/kg	° Administration of lycopene reduced serum glucose and enhanced serum insulin levels.	[63]
STZ-induced diabetic rats	20 mg/kg	° Lycopene protects diabetic nephropathy and ameliorates renal function.	[64]
STZ-induced diabetic nephropathy in rats	20 mg/kg	° Lycopene treatment showed protective effect against diabetic nephropathy.	[65]
STZ-induced diabetic rats	0, 20, 40 and 2 mg/kg	° Lycopene decreased blood glucose concentration. ° The serum insulin level increased compared with diabetic control animals.	[66]
STZ-induced diabetics in rats	10 mg/kg	° Lycopene reduced diabetic plasma glucose level; ° Lycopene decreased lipid peroxidation as well as NO in plasma.	[67]
STZ-induced diabetics in rats	4 mg/kg	° The blood glucose levels were decreased by supplementation of lycopene.	[68]
STZ-induced diabetic rats	10, 30, 60 mg/kg b.w	° Reduced in FBG levels.	[69]

**Table 9:** Summary of clinical studies evaluating the therapeutic effects of lycopene supplementation or lycopene-enriched formulations in different patient populations. The table highlights study design, cohort characteristics, sample size, mode of lycopene administration, and the main clinical outcomes associated with lycopene treatment.

Study Design	Cohort	Sample Size	Lycopene Administration	Outcomes	Refs.
Randomized controlled trial	Postmenopausal women	33	Lycopene intake into four groups ranging from 1.76 to 7.35 mg/day	° Lycopene has shown to reduce oxidative stress and lower the levels of bone turnover markers in postmenopausal women, and it may play a valuable role in reducing the risk of osteoporosis.	[209]
Clinical study	Postmenopausal women	39	Lycopene-rich tomato sauce 3.9 mg/day	° A substantial bone density loss was not noticed in women taking the tomato sauce. ° Tomato sauce intake caused a greater bone alkaline phosphatase decrease.	[211]
Double-blind, randomized, placebo-controlled clinical trial	Patient with burning mouth syndrome	60	Lycopene-enriched extra virgin olive oil applied as a spray to the mouth	° Topical lycopene-enriched virgin olive oil is safe and an effectively similar way in which the placebo is used for treating BMS patients.	[212]
Double-blind, randomized, placebo-controlled clinical trial	Patients’ xerostomia	60	Lycopene-enriched EVOO applied as a spray to the mouth	° The topical application of lycopene and its placebo counterpart improved xerostomia.	[213]
Randomized, double-blind, placebo-controlled clinical trial	Infertile men	44	25 mg of lycopene	° Lycopene supplements improve sperm parameters and oxidative stress biomarkers.	[214]
Placebo-controlled double-blind randomized clinical trial	Postmenopausal women	108	Soft gel of LycoRed withs 2 mg as lycopene and phytonutrients	° Lycopene supplementation in menopausal women confers protection from osteoporosis.	[215]

## Data Availability

No new data were generated or analyzed during this study; therefore, data sharing is not applicable.

## Abbreviations

5-FU	5-Fluorouracil
Bax	Bcl-2-associated X protein
BPH	Benign prostatic hyperplasia
CAT	Catalase
CCl4	Carbon tetrachloride
COX-2	Cyclooxygenase-2
CP	Cisplatin
CVDs	Cardiovascular diseases
DPPC	Dipalmitoyl-phosphatidylcholine
GEN	Gentamicin
GSH-Px	Glutathione peroxidase
GST	Glutathione S-transferase
HFD	High fat diet
HSV-1	Herpes simplex virus type 1
IL-6	Interleukin-6
ISO	Isoproterenol
LDH	Lactate dehydrogenase
LPO	Lipid peroxidation
LPS	Lipopolysaccharide
MDA	Malondialdehyde
MMP-2	Matrix metalloproteinase-2
MPTP	1-Methyl-4-phenyl-1,2,3,6-tetrahydropyridine
MRSA	Methicillin-resistant *Staphylococcus aureus*
PD	Parkinson’s disease
PPA	Propionic acid
PTZ	Pentylenetetrazol
TD	Tramadol
TNF	Tumor necrosis factor
Txn-1	Thioredoxin-1
UC	Ulcerative colitis
VEGF	Vascular endothelial growth factor
BMS	Burning mouth syndrome
PON1	Paraoxonase 1
NOAEL	The no-observed-adverse-effect level
