# Correction: Almatroodi et al. Exploring the Therapeutic Potential of Lycopene: Mechanisms, Biological Activities, and Health Benefits. *Int*. *J*. *Mol*. *Sci*. 2026, *27*, 5386

**DOI:** 10.3390/ijms27208949

**Published:** 2026-10-09

**Authors:** Saleh A. Almatroodi, Mohammad Alshebremi, Arshad Husain Rahmani

**Affiliations:** Department of Medical Laboratories, College of Applied Medical Sciences, https://ror.org/01wsfe280Qassim University, Buraydah 51452, Saudi Arabia; smtrody@qu.edu.sa (S.A.A.); m.alshebremi@qu.edu.sa (M.A.)

## References

The following references [181] have been retracted and should be removed from the original publication [1]:

[181] El-Masry, T.A.; El-Nagar, M.M.F.; El Mahdy, N.A.; Alherz, F.A.; Taher, R.; Osman, E.Y. Potential Antitumor Activity of Combined Lycopene and Sorafenib against Solid Ehrlich Carcinoma via Targeting Autophagy and Apoptosis and Suppressing Proliferation. *Pharmaceuticals*
**2024**, *17*, 527.

With this correction, the order of some references has been adjusted accordingly.

In order to maintain accuracy and precision, a correction has been made to the sentence containing the original references [181] in Section 5, ii. Combination with Chemotherapeutic Agents, paragraph number 2:

Moreover, combination treatment reduced inflammation and enhanced apoptosis, as observed by the tumor’s weight and decreased volume.

In addition, reference [181] was removed from the “Sorafenib” entry in row 10 of Table 7.

The authors state that the scientific conclusions are unaffected. The corrections were approved by the Academic Editor. The original publication has also been updated.
